# Structure-mechanism-based engineering of chemical regulators targeting distinct pathological factors in Alzheimer's disease

**DOI:** 10.1038/ncomms13115

**Published:** 2016-10-13

**Authors:** Michael W. Beck, Jeffrey S. Derrick, Richard A. Kerr, Shin Bi Oh, Woo Jong Cho, Shin Jung C. Lee, Yonghwan Ji, Jiyeon Han, Zahra Aliakbar Tehrani, Nayoung Suh, Sujeong Kim, Scott D. Larsen, Kwang S. Kim, Joo-Yong Lee, Brandon T. Ruotolo, Mi Hee Lim

**Affiliations:** 1 Department of Chemistry, Ulsan National Institute of Science and Technology (UNIST), Ulsan 44919, Republic of Korea; 2 Department of Chemistry, University of Michigan, Ann Arbor, Michigan 48109, USA; 3 Asan Institute for Life Sciences, Asan Medical Center, Seoul 05505, Republic of Korea; 4 School of Life Sciences, Ulsan National Institute of Science and Technology (UNIST), Ulsan 44919, Republic of Korea; 5 Department of Medicinal Chemistry, University of Michigan, Ann Arbor, Michigan 48109, USA; 6 Department of Convergence Medicine, University of Ulsan College of Medicine, Seoul 05505, Republic of Korea

## Abstract

The absence of effective therapeutics against Alzheimer's disease (AD) is a result of the limited understanding of its multifaceted aetiology. Because of the lack of chemical tools to identify pathological factors, investigations into AD pathogenesis have also been insubstantial. Here we report chemical regulators that demonstrate distinct specificity towards targets linked to AD pathology, including metals, amyloid-β (Aβ), metal–Aβ, reactive oxygen species, and free organic radicals. We obtained these chemical regulators through a rational structure-mechanism-based design strategy. We performed structural variations of small molecules for fine-tuning their electronic properties, such as ionization potentials and mechanistic pathways for reactivity towards different targets. We established *in vitro* and/or *in vivo* efficacies of the regulators for modulating their targets' reactivities, ameliorating toxicity, reducing amyloid pathology, and improving cognitive deficits. Our chemical tools show promise for deciphering AD pathogenesis and discovering effective drugs.

## Abstract (web summary)

To advance our understanding of pathological features associated with Alzheimer's disease (AD), chemical tools with distinct specificity towards AD targets would be valuable. Here the authors used a structure-mechanism-based design strategy to obtain small molecules as chemical regulators for distinct pathological factors linked to AD pathology.

## Graphical abstract

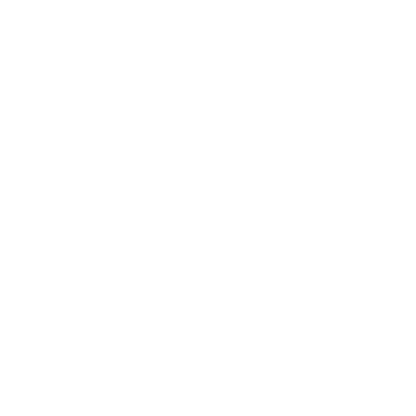

Multifactorial disease pathology is a unifying theme of Alzheimer's disease (AD), the most common of all neurodegenerative diseases^1^,^2^,^3^. Misfolded protein aggregate formation, metal ion dyshomeostasis and oxidative stress are some of the many factors that have been implicated in AD onset and progression^1^,^2^,^3^,^4^,^5^,^6^,^7^,^8^,^9^,^10^,^11^,^12^,^13^. The inter-relationships between these individual facets further impede our ability to fully comprehend the disease mechanisms and thus identify the most upstream causative elements. For example, the production of metal–protein complexes can subsequently promote the misfolding and stabilization of abnormal and toxic protein conformations, along with the generation of reactive oxygen species (ROS) through Fenton-like chemistry (in the case of redox-active metals)^1^,^2^,^3^,^6^,^7^,^8^,^9^,^10^,^11^,^13^,^14^,^15^,^16^,^17^,^18^,^19^. Thus, to address the inherent complexities of AD, novel strategies must be available for determination of pathological factors (for example, misfolded proteins, metal ions and ROS) and elucidation of their individual or inter-related roles in the disease.

The rational design of chemical tools to specifically probe individual pathological facets of interest and modulate their activities *in vitro* is valuable for providing a molecular-level understanding of AD pathogenesis. Such molecular-level findings cannot be easily achieved from other commonly used *in vivo* or genetic approaches that are further limited by the absence of model systems that completely mimic human AD^2^,^3^,^20^,^21^,^22^,^23^,^24^,^25^. Particularly, chemical tools with the ability to specifically interact with different targets of interest must be devised. To date, the development of tools to investigate the involvement of metal-free Aβ, metal–Aβ and ROS in AD pathology has been impaired due to a few key reasons. First, there have been a limited number of suitable molecular frameworks that can be used as a starting point for rational structure-based design and possess the biological properties required (for example, blood–brain barrier (BBB) permeability, water solubility)^2^,^20^,^21^,^22^,^23^,^24^,^25^,^26^. Furthermore, there is little understanding of how slight structural alterations to existing Aβ-imaging frameworks vary tools' reactivity and target specificity. Without such knowledge that could eventually be applied to establish the criteria for newly designed tools, researchers must rely on costly, time-consuming high-throughput screening methods to discover effective molecules. Finally, there are limited reports that describe the modes of action between chemical tools and targets of interest at the molecular level^22^,^24^,^25^,^27^,^28^,^29^. Correct applications of chemical tools cannot be pursued without detailed information on how the molecule and protein interact.

Herein, we report chemical tools (particularly, chemical regulators) designed based on a structure-mechanism-based concept. This design principle exemplifies that different properties (for example, metal binding, Aβ interaction and ionization potentials (IPs)) of small molecules afford chemical tools that have specific reactivity with distinct pathological targets associated with AD (that is, metals, metal-free and metal-bound Aβ, ROS and free organic radicals; Fig. 1a) through disparate mechanistic pathways. Such chemical regulators were readily obtained through slight structural variations to a parent framework (Fig. 1a). On the basis of biochemical, biophysical and computational approaches, our chemical regulators are indicated to modulate metal-free or metal-bound Aβ aggregation *in vitro* to different degrees through multiple structure-dependent mechanisms (for example, complex or covalent adduct formation with peptides and peptide modifications). In addition, structural modifications to the framework are presented to tune regulatory activities towards ROS and free organic radicals, as predicted by their IPs. Furthermore, the *in vivo* efficacy for our chemical regulator (**1**, Fig. 1a) was confirmed in an AD mouse model. Overall, our studies demonstrate the structure-mechanism-based development of chemical tools capable of targeting and controlling individual or inter-related AD pathological factors via minor structural modifications to a parent entity.

## Results

### Rational design and characterization of small molecules

**Figure 1:**
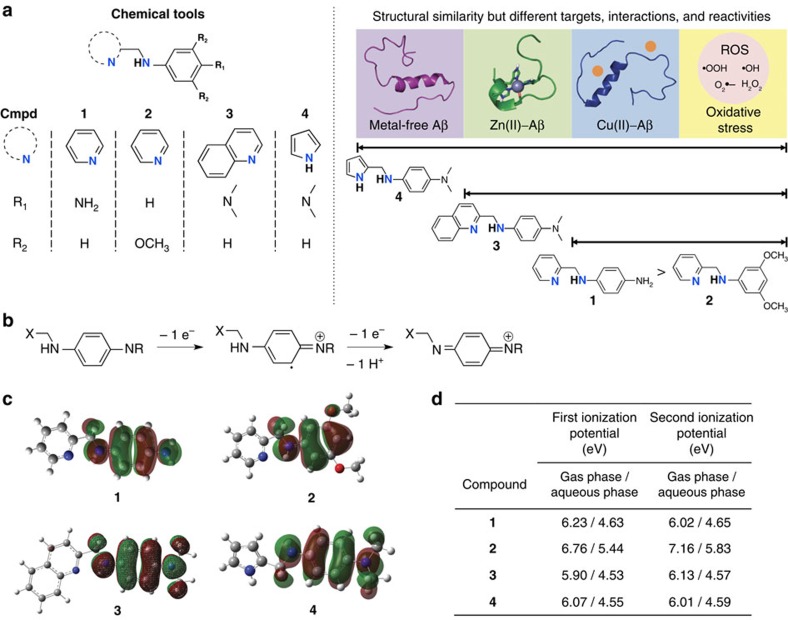
Targets associated with AD and ionization potentials of chemical tools (1–4). (**a**) Structures of **1–4** [**1**, *N*^1^-(pyridin-2-ylmethyl)benzene-1,4-diamine; **2**, 3,5-dimethoxy-*N*-(pyridin-2-ylmethyl)aniline; **3**, *N*^1^,*N*^1^-dimethyl-*N*^4^-(quinolin-2-ylmethyl)benzene-1,4-diamine; **4**, *N*^1^-((1*H*-pyrrol-2-yl)methyl)-*N*^4^,*N*^4^-dimethylbenzene-1,4-diamine] and their targets (metal-free Aβ, Cu(II)–Aβ, Zn(II)–Aβ and ROS). (**b**) Scheme of the oxidation of *p*-phenylenediamines. (**c**) Isosurface plot of SOMOs of cationic radicals of **1**–**4** with an isovalue of 0.02 au (red: O; blue: N; grey: C; white: H). (**d**) Calculated ionization potentials for **1**–**4** for the first and second processes depicted in **b** in both the gas and aqueous phases.

Four small molecules (**1–4**; Fig. 1a) with similar chemical structures were rationally designed to interact with and regulate distinct targets (that is, metals, metal-free Aβ, metal (Cu(II) or Zn(II))–Aβ, ROS, free organic radicals; Fig. 1a) by incorporating structural moieties for metal binding and Aβ interaction into a framework, along with potential antioxidant activity and BBB permeability. These compounds were obtained and used after purification (Supplementary Methods). For metal chelation, the molecules have two nitrogen (N) donor atoms provided by structural portions of 2-picolylamine (for **1** and **2**), quinolin-2-ylmethanamine (for **3**), or (1*H*-pyrrol-2-yl)methylamine (for **4**) (Fig. 1a). Moreover, for different Aβ interacting properties, the structures were varied by installing amino (for **1**), 3,5-dimethoxy (for **2**), or dimethylamino (for **3** and **4**) functionalities (Fig. 1a).

Furthermore, tuning the electronic properties, such as IPs, was considered in our molecule design for their antioxidant activity. The first and second adiabatic IPs (IP_1_ and IP_2_) for **1–4** were calculated in both the gas and aqueous phases (Fig. 1b-d). These studies present that **2**, relative to **1**, **3** and **4**, has higher IPs. This is because the unpaired electron in the cation radical species is computed to be mainly stabilized via *π*-delocalization on the benzene ring, *σ*-conjugation on the *para*-substituted position, and combinations of *π*-delocalization and *σ*-conjugation features. As shown in Fig. 1c, the singly occupied molecular orbitals (SOMOs) of the cationic radicals of **1**, **3** and **4** are located on the benzene ring and the *para*-diamino substitution (via *π*-delocalization and *σ*-conjugation), whereas the radical on **2** is mainly located at the benzene ring. In addition, the *para*-substituted groups of **1**, **3** and **4** can be mixed into the SOMOs, but *meta*-substitutions in **2** cannot. As a result, the amino group in **1**, **3** or **4** raises the level of SOMOs by its mesomeric effect, in contrast to the methoxy groups in **2** lowering the SOMOs by the inductive effect. Thus, **2** is least likely to undergo oxidation. Lastly, the BBB permeability of **1**–**4** was examined. First, adherence to Lipinski's rules and calculated logBB values were confirmed. All calculated values suggest that **1**–**4** could penetrate the BBB, along with the experimental results (permeability values, –log*P*_e_) from an *in vitro* parallel artificial membrane permeability assay adapted for the BBB (Supplementary Table 1).

### Modulation of metal-free and metal-induced Aβ aggregation

**Figure 2:**
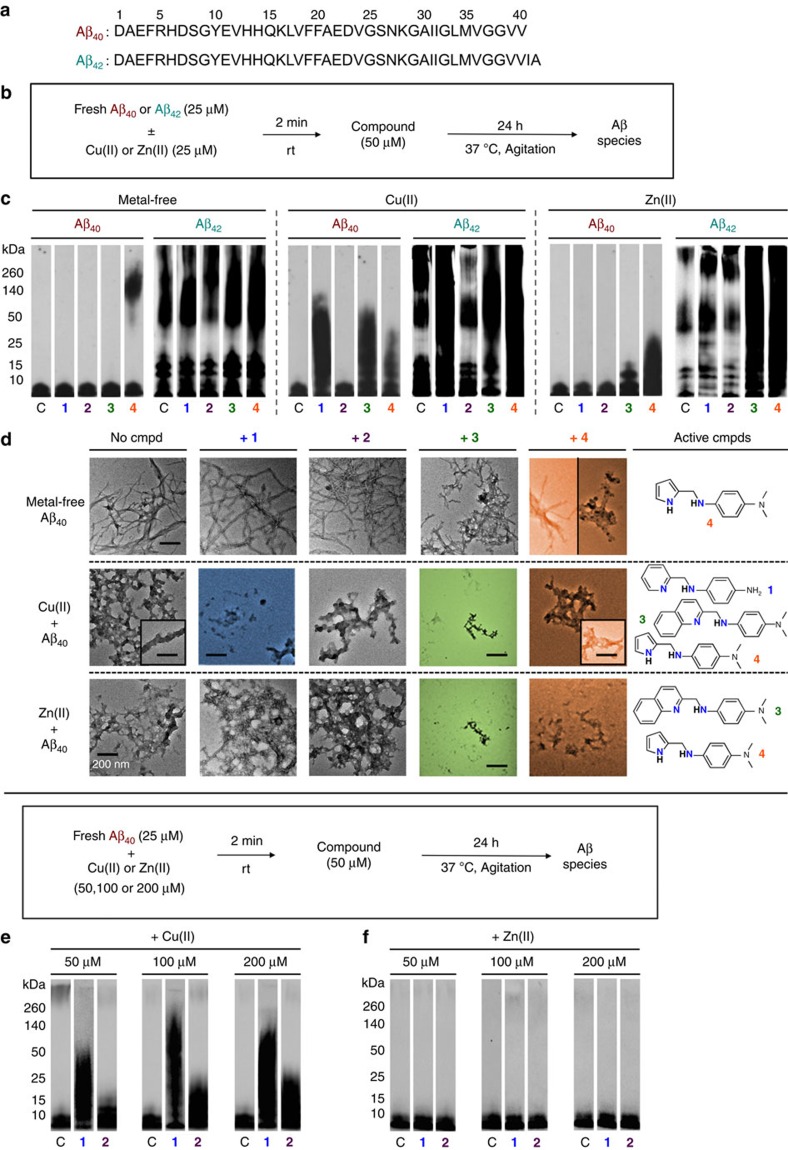
Effects of the compounds on metal-free and metal-induced Aβ aggregation. (**a**) Amino acid sequences of Aβ_40_ and Aβ_42_. (**b**) Scheme of the inhibition experiment: freshly prepared Aβ (25 μM) in the presence or absence of Cu(II) (blue, 25 μM) or Zn(II) (green, 25 μM) was mixed without (lane C) or with compounds (**1**–**4**; 50 μM) and incubated at 37 °C with constant agitation for 24 h. (**c**) Gel/western blot analysis of the MW distribution of the resulting Aβ_40_ and Aβ_42_ species using anti-Aβ antibody (6E10). (**d**) Morphologies of the Aβ_40_ aggregates after treatment with **1**–**4** as observed using TEM (scale bar, 200 nm). (**e**,**f**) Ability of **2** (50 μM), compared with **1**, to modulate the aggregation pathway of Aβ_40_ (25 μM) in the presence of various amounts of Cu(II) (left) or Zn(II) (right) (left, 50 μM; middle, 100 μM; right, 200 μM), monitored by gel/western blots.

The ability of **1**–**4** to control the aggregation of both metal-free Aβ and metal-Aβ in inhibition (Fig. 2) and disaggregation experiments (Supplementary Fig. 1) was evaluated using the two major Aβ isoforms (Aβ_40_/Aβ_42_)^1^,^2^,^3^,^6^,^16^ found in the AD-affected brain. Molecular weight (MW) distributions and morphologies of the resulting Aβ species were determined by gel electrophoresis followed by western blotting (gel/western blot) and transmission electron microscopy (TEM). Generally, under the experimental conditions employed herein, compound-free Aβ samples with and without metal ions assemble into a distribution of large aggregates that are too big to penetrate into the gel matrix, which yields very little smearing in the gel/western blots, but they can be visualized by TEM. The administration of compounds, able to interact with Aβ, inhibit the formation of high MW aggregates, and/or disassemble preformed aggregates, typically generates smaller-sized Aβ species that can enter into the gel and produce a substantial amount of streaking compared with the samples containing Aβ only.

The alteration of metal-free Aβ aggregation by **1**–**4** was first studied. Noticeable influence of **1**–**3** on metal-free Aβ aggregation was not observed (Fig. 2b–d, Supplementary Figs 1 and 2). Changing the pyridine (from **1** and **2**) and quinoline (from **3**) moieties to a pyrrole (from **4**), however, had a pronounced influence on metal-free Aβ aggregation. Treatment of Aβ_40_ with **4** produced aggregates that were >50 kDa in both inhibition and disaggregation samples, while increased species (MW≤50 kDa) were detected in the case of Aβ_42_ by gel/western blots, which was more evident in experiments of inhibition over disaggregation (Fig. 2c and Supplementary Fig. 1b,d). Additionally, in the inhibition and disaggregation experiments using Aβ_40_, smaller and more amorphous species were indicated by TEM (Fig. 2d and Supplementary Fig. 1c); however, these changes were less noticeable in the samples containing Aβ_42_ (Supplementary Figs 1e and 2).

Next, Cu(II)- and Zn(II)-induced aggregation was detected upon treatment of **1**–**4**. **1** demonstrated a capacity to only redirect Cu(II)-promoted Aβ_40_ and Aβ_42_ aggregation (Fig. 2c and Supplementary Fig. 1b,d). TEM revealed the presence of less structured forms of aggregates (Fig. 2d and Supplementary Figs 1c,e and 2). **1** did not show any noticeable activity towards Zn(II)-mediated Aβ_40_/Aβ_42_ aggregation even at higher Zn(II) concentrations (Fig. 2f). Compound **2** was found to only have a modulating activity at higher Cu(II) concentrations, indicated by gel/western blot and TEM (Fig. 2c–e and Supplementary Figs 1b–e and 2).

The Cu(II)-specific activity of **1** and **2** is contrasted to **3** which modulates Aβ aggregation involved by both Cu(II) and Zn(II) (Fig. 2c,d and Supplementary Fig. 1b–e). **4** was also found in the gel/western blots to redirect both Cu(II)- and Zn(II)-induced Aβ aggregation, indicating the production of smaller less structured fibrils, which was visualized by TEM (Fig. 2c,d and Supplementary Figs 1b–e and 2). Overall, the results from gel/western blots and TEM demonstrate that by varying the structures of compounds we are able to change their potential to interact with metal-free Aβ and/or metal–Aβ (both Cu(II)–Aβ and Zn(II)–Aβ; only Cu(II)–Aβ) and divert the aggregation pathways to form potentially nontoxic off-pathway species^27^,^28^,^29^.

### Mediation of oxidative stress

**Figure 3:**
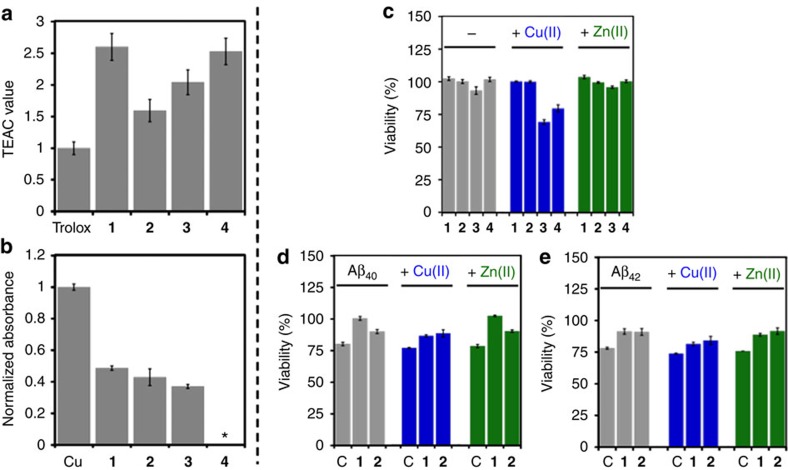
Biological activities of 1–4. (**a**) Antioxidant activity of **1**–**4** as evaluated by the Trolox equivalent antioxidant capacity (TEAC) assay employing human neuroblastoma SK-N-BE(2)-M17 (M17) cell lysates. All values are relative to a vitamin E analogue, Trolox (6-hydroxy-2,5,7,8-tetramethylchroman-2-carboxylic acid). (**b**) Inhibition of Cu(I/II)-triggered Fenton-like ROS production by **1**–**3** (125 μM) as measured by the 2-deoxyribose assay ([Cu(II)]=10 μM). * **4** was not tested due to limited solubility in the assay buffer. (**c**) Toxicity of **1**–**4** (20 μM) to M17 cells with and without metal ions for 24 h. Cytotoxicity data of **1**–**4** (different concentrations and incubation time points) are presented in Supplementary Fig. 3. (**d**,**e**) Ability of **1** and **2** (20 μM) to mediate the toxicity of (**d**) Aβ_40_ (20 μM) and (**e**) Aβ_42_ (20 μM) in the absence (left, grey) and presence of CuCl_2_ (middle, blue; 20 μM) or ZnCl_2_ (right, green; 20 μM) in M17 cells for 24 h. ‘C' indicates the samples untreated with compounds. Viability of cells (%) was calculated relative to that of cells incubated only with 1% v/v DMSO. Error bars represent the standard deviation (s.d.) from three independent experiments (*P*<0.05).

The capability of **1**–**4** to scavenge free organic radicals was explored in cell lysates using the Trolox equivalence antioxidant capacity (TEAC) assay used for testing their aptness to quench the cationic organic radical of 2,2′-azino-bis(3-ethylbenzothiazoline-6-sulfonic acid (ABTS) compared with the known antioxidant, Trolox, a water-soluble analogue of vitamin E^25^,^28^,^29^. All compounds showed a greater free radical scavenging ability than Trolox (Fig. 3a). Specifically, **1**, **3** and **4** containing the *p*-amine substitutions had a greater capacity than **2** with the 3,5-dimethoxy substitution. This suggests that an electron donating *p*-amine functionality on the backbone of molecules can increase their ability to be oxidized and act as an antioxidant, consistent with calculated IP values (Fig. 1b–d, vide supra).

Moreover, the inhibitory activity of **1**–**4** towards the generation of hydroxyl radicals by Cu(I/II) through Fenton-like reactions was evaluated using the 2-deoxyribose assay^30^. **1**–**3** could reduce the generation of hydroxyl radicals by approximately half (Fig. 3b; note that **4** was not tested due to poor solubility under the assay conditions). Together, our frameworks with different aniline groups are shown to diminish the presence and production of ROS, which could reduce oxidative stress.

### Regulation of metal-free and metal-treated Aβ cytotoxicity

The toxicity of compounds and their ability to alleviate metal-free/-bound Aβ toxicity were analysed employing human neuroblastoma SK-N-BE(2)-M17 (M17) cells. **1** and **2** were indicated to be nontoxic in the M17 cells with and without Cu(II) and Zn(II) at the tested concentrations (up to 20 μM) that did not show interference with the analysis window of the cell viability assay (Fig. 3c and Supplementary Fig. 3). **3** and **4**, however, decreased cell viability in the presence of Cu(II) (*∼*70–80% viability at 20 μM of compounds) for 24 h as well as in the absence of Cu(II) for 48 and 72 h (up to *∼*60% viability). In addition, when **1** or **2** was co-incubated with Aβ_40_/Aβ_42_ in the absence and presence of Cu(II) and Zn(II), the toxicity induced by metal–Aβ was diminished (Fig. 3d,e). Note that **3** and **4** were not studied with Aβ species due to its noticeable toxicity with Cu(II) in the absence of peptides. These results may stem from redirection of the aggregation pathways of Aβ_40_ and Aβ_42_ to produce off-pathway species as observed in the gel/western blot and TEM studies that are less toxic than on-pathway Aβ aggregates^27^,^28^,^29^, along with their capability of modulating oxidative stress (vide supra).

### *In vivo* efficacy

The *in vivo* efficacy of **1** was evaluated using the 5 × FAD AD mouse model due to its specificity for mediating Cu(II)–Aβ aggregation and toxicity *in vitro* and in living cells, along with its solubility in aqueous media (the HCl salt of **1**, *∼*800 mg ml^−1^ in water) and regulatory activity towards oxidative stress (vide supra). We refrained from analysing **2** *in vivo* because it possessed the same target specificity as **1** (that is, specificity for Cu(II)–Aβ), but it requires elevated concentrations of Cu(II) and had much lower degrees of water solubility and antioxidant activity relative to **1**. **3** and **4** were also not selected for *in vivo* analysis due to their lower cell viability (with respect to **1**) with and without Cu(II) (vide supra).

**Figure 4:**
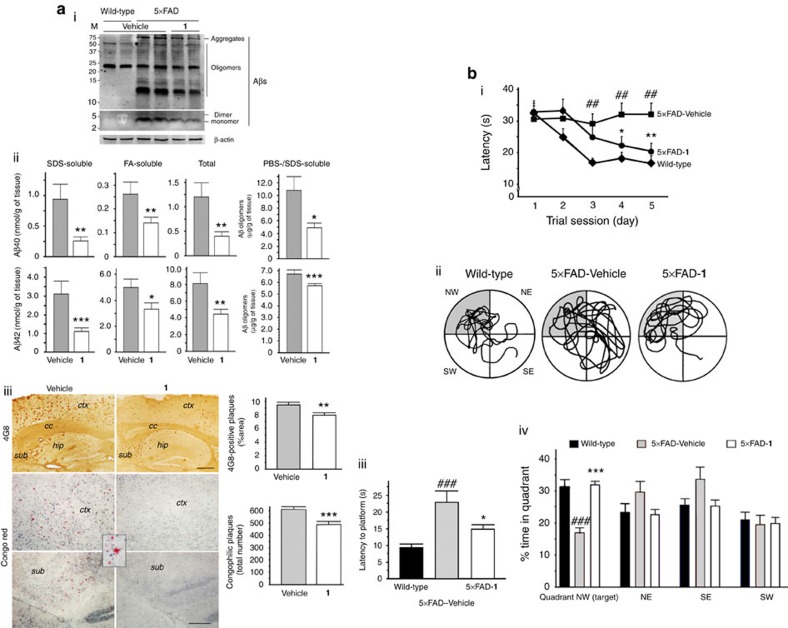
*In vivo* efficacy of 1 against amyloid pathology and cognitive defects. (**a**) Determination of Aβ levels (**i**,**ii**) and loads of 4G8-immunoreactive (**iii**, top, brown) and Congo red-positive (**iii**, bottom, red) amyloid plaques in the brains of 5 × FAD mice after 30-day treatment with **1**. ELISA analyses were performed in triplicate per sample to quantify Aβ oligomers or aggregates as well as SDS-soluble, FA-soluble, and total Aβ_40_ and Aβ_42_ (**ii**) (*n*=7 and 10 for vehicle- and **1**-treated 5 × FAD mice, respectively). Aβ-immunohistochemistry (brown) or Congo red staining (red, inset) was conducted in the brains of vehicle- or **1**-treated 5 × FAD mice (**iii**). The area of 4G8-immunoreactive amyloid deposits or the total number of congophilic amyloid plaques in the same cortical region of interest was measured in five brain sections taken from each animal. Representative microscopic images of cortical or subiculum area in the Congo red-stained (red) brain sections of 5 × FAD mice. ctx, cortex; hip, hippocampus, cc; corpus callosum, sub; subiculum. Scale bars=100 μm. All bars denote mean±s.e.m. (*n*=14 and 17 for vehicle- and **1**-treated 5 × FAD mice, respectively). *P<0.05, **P<0.01, or ***P<0.001 by unpaired two-tail *t*-test. (**b**) Enhancement of cognitive performance by **1** in the 5 × FAD mice. Using the Morris water maze (MWM) test, spatial learning and memory performance was compared between 5 × FAD and their littermate wild-type mice after 30-day treatment with vehicle or **1**. (**i**) The escape latency time was measured every day for 5 days from the day of the 30th drug treatment. (**ii**–**iv**) The probe trials were conducted at 3 h after the final trial of the MWM test. **ii**, The images depict the representative traces of mice to search for the escape platform in the water maze for 60 s. (**iii**,**iv**) Bars denote the time when they reached the platform area (**iii**) and stay in the target quadrant (NW, grey area in **ii**; **iv**). The statistical comparisons were performed between 5 × FAD and their wild-type littermate mice with vehicle (#), or between vehicle and **1** treatment in 5 × FAD mice (*). All values denote mean±s.e.m. (*n*=14 and 17 for vehicle- and **1**-treated 5 × FAD mice, respectively; *n*=17 for vehicle-treated wild-type mice). *P<0.05, **^,##^*P*<0.01 or ***,^###^*P*<0.01.

**1** was administered to the 3-month-old 5 × FAD mice at a daily dosage of 1 mg kg^−1^ by an intraperitoneal injection every day for 30 days. All mice tolerated the repeated treatments and experienced no gross changes in the post-necropsy evaluation. There was no difference in body weight between **1**- and vehicle-treated 5 × FAD mice throughout the drug administration period (Supplementary Table 2). Daily repeated administrations of **1** significantly lessened the amyloid pathology in the brains of 5 × FAD mice. The total Aβ level, composed of phosphate buffered saline (PBS)-, SDS- and formic acid (FA)-soluble Aβ_40_/Aβ_42_, became significantly lower than that in the vehicle-treated 5 × FAD mice (Fig. 4a(ii)). Amounts of Aβ oligomers and aggregates also decreased in the **1**-treated 5 × FAD mice (Fig. 4a(i,ii)). In addition, **1** reduced the loads of Aβ deposits in the brain, as determined by the evaluation of the 4G8-immunoreactive or congophilic Aβ plaques (Fig. 4a(iii)).

Moreover, the Morris water maze test was conducted to evaluate the cognitive functions of 5 × FAD mice in response to the persistent treatment with **1**. During the trial trainings, the **1**-treated 5 × FAD mice could find the hidden escape platform faster than the vehicle-treated 5 × FAD mice, which was comparable to the performance of wild-type mice at the same age (Fig. 4b(i)). The probe trials also showed significant improvement of the long-term spatial memory in the **1**-treated animals (Fig. 4b(ii,iii)). Therefore, **1** could produce beneficial effects to prevent or reverse cognitive deficits as well as amyloid pathology in 5 × FAD mice. The alleviation of AD symptoms and pathology, which arises from mediating Cu(II)–Aβ aggregation and toxicity by treatment with **1**, is suggested to be effective as indicated by the previously reported studies using copper ionophores (for example, clioquinol and PBT2) in transgenic AD mice^31^,^32^.

### Characterization of solution species

Studies of the species present in solution indicated that **1** and **2** were stable over 5 h (Supplementary Fig. 4a–h). **3** showed changes in its Ultraviolet-Visible (UV-vis) spectra after 5 h of incubation occurring at a very slow rate (Supplementary Fig. 4i and Supplementary Table 3). Electrospray ionization mass spectrometry (ESI–MS) studies, however, exhibited no change in the major peak at 278 *m/*z corresponding to the [M+H]^+^ ion after 5 h of incubation supporting the stability of **3** (Supplementary Fig. 4j–l). Moreover, **4** was unstable with a half-life of *∼*40 min (Supplementary Table 3). After 5 h of incubation, ESI–MS identified a peak at 137 *m/z* corresponding to [M + H]^+^ of *N*,*N*-dimethyl-*p*-phenylenediamine (**DMPD**)^29^ but other possible degradation products of **4** could not be identified (Supplementary Fig. 4m–p). This could be due to the reported propensity of the pyrrole moiety to polymerize as possibly evidenced by unidentified peaks at 288 and 297 *m/z* (Supplementary Fig. 4o)^33^,^34^. **DMPD** was recently reported to interact with both metal-free Aβ and metal–Aβ and redirect their self-assembly routes to form off-pathway aggregates, suggested to be less toxic^29^.

The stability in the presence of Cu(II) was also determined. UV-vis and ESI–MS studies indicated that **1** initially formed CuL_2_ complexes (L=ligand; [M + H]^+^=460 *m/z*), with the half-life being *∼*5 min, followed by the generation of one-electron ([M]^+^) or two-electron oxidation ([M + H]^+^) products at 198 *m/z* (Supplementary Fig. 5a–d and Supplementary Table 3). These types of oxidation products are well defined in the literature for unsubstituted and substituted *p-*phenylenediamine derivatives with an oxidant^35^,^36^,^37^,^38^. **3** also presented UV-vis spectral changes similar to **1** with the decay of the peak attributed to Cu(II) binding at *∼*450 nm (half-life *∼*50 min) corresponding to the growth of a new peak at *∼*550 nm comparable to the reported one-electron oxidation products of *p-*phenylenediamine derivatives (Supplementary Fig. 5i). ESI–MS studies confirmed the initial formation of a CuL_2_ complex of **3** which was observed to degrade into the metal-free two-electron oxidized form (Supplementary Fig. 5j–l). Note that other species were detected by ESI–MS after 5 h of incubation, possibly from further degradation of **3**. The identity of these species will be the subject of future studies.

In the case of **2**, the addition of Cu(II) created a new peak at *∼*375 nm that did not dissipate over 5 h (Supplementary Fig. 5e). ESI–MS identified an ion at 588 *m/z* corresponding to the CuL_2_ complex ([M + K]^+^; Supplementary Fig. 5f-h). Different from **1** and **3**, **2** generated a complex with Cu(II) without any further transformations over the course of 5 h; thus, the binding affinity of **2** for Cu(II) was estimated using UV–vis variable-pH titrations. The presence of a 1:1 complex was observed with an approximate disassociation constant in the micromolar range (Supplementary Fig. 6). This low binding affinity is likely to be too weak to interact with the low micromolar to high picomolar (*∼*10^−7^–10^−11^ M)^1^,^16^,^39^ binding affinity for the first Cu(II) binding site of Aβ. Interaction is possible, however, with the second metal binding site in Aβ which has a weaker affinity of *∼*10^−5^ M for Cu(II)^1^,^16^,^39^. This is consistent with the results from the gel/western blots where at least two equivalents of Cu(II) are required for activity (Fig. 2e), suggesting that **2** can only interact with Aβ when both metal binding sites are metalated (vide supra).

Lastly, the addition of Cu(II) to **4** resulted in intense double peaks at *∼*510 and 550 nm within 1 min, similar to the previously reported spectra of the cationic radical of **DMPD** (Supplementary Fig. 5m)^29^. ESI–MS displayed the peaks at 137 and 216 *m*/*z* corresponding to [**DMPD**+H]^+^ and [**4**+H]^+^, respectively (Supplementary Fig. 5n,o; note that longer incubations led to precipitation thus limiting analysis). This suggests that **DMPD** may be responsible for the activity of **4** towards Aβ (ref. 29).

### Mass spectrometric studies for Aβ and metal–Aβ interactions

**Figure 5:**
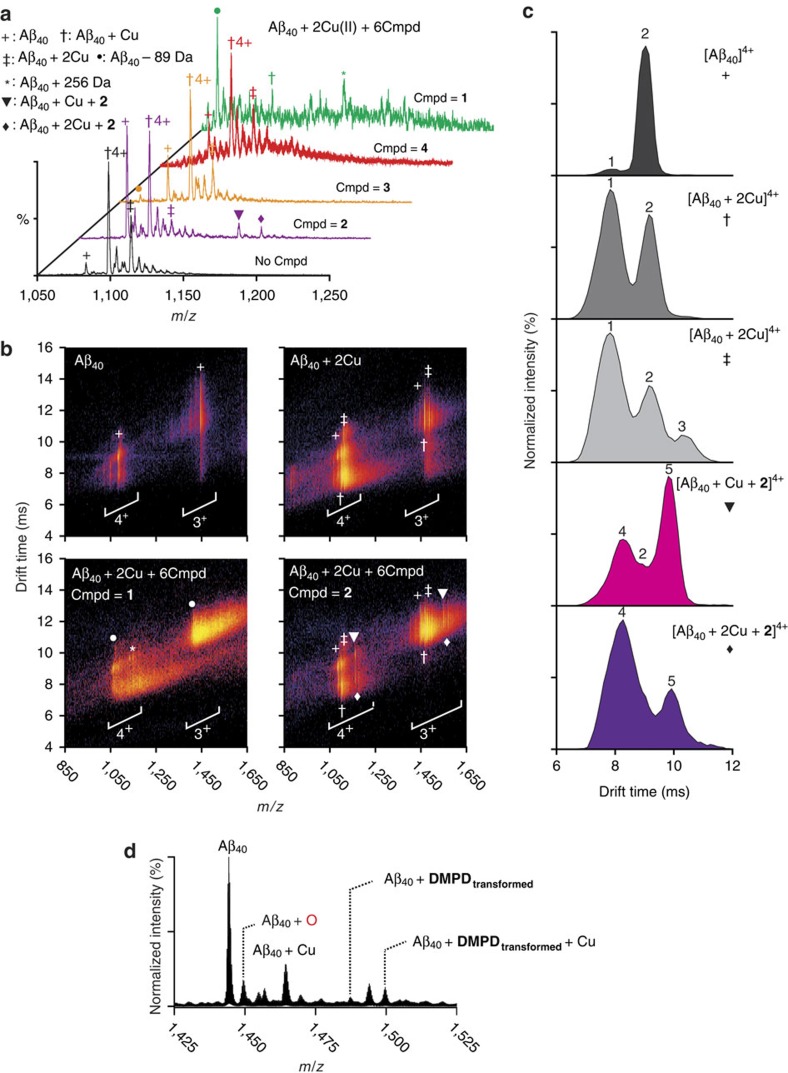
ESI–MS and IM–MS analyses of 1–4 in the presence or absence of Cu(II). (**a**) Metal-dependent interactions of **1**–**4** with monomeric Aβ_40_. Both **1** and **3** promote the formation of Aβ mass loss product 89 Da lighter than the metal-free peptide (●), consistent with previously published data^25^. Incubations of Aβ with **1** were additionally shown to produce an adduct consistent with a mass gain of 256 Da compared with the intact, unmodified, peptide (*). **2** forms stable ternary complexes with Aβ_40_ and Cu(II), existing in two different stoichiometries that contain either one or two equivalents of the metal (▾ and ♦, respectively). Conditions for (**a**): [Aβ_40_]=18 μM; [copper(II) acetate]=40 μM; [**1**–**4**]=120 μM; 100 mM ammonium acetate (pH 7.5) with 1% v/v DMSO; 37 °C; 30 min incubation. (**b**) IM drift time *versus m*/*z* plots comparing the data acquired for **1** (bottom left) and **2** (bottom right) against Cu(II)-bound Aβ_40_ (top right) and Cu-free Aβ_40_ (top left). (**c**) Arrival time distribution data extracted from the plots which provide that these stable binding interactions support altered distributions upon binding to **2** when compared with the metal-free and Cu-bound Aβ_40_ complexes (collision cross section (CCS) data presented in Supplementary Table 4). (**d**) ESI–MS spectra of Aβ_40_ with **4** in the presence of Cu(II) under different conditions from (**a**). Aβ–**DMPD**_**transformed**_ complexes and oxidized Aβ_40_ were found when Cu(II) was present. The different positive charge states (with H^+^ and Na^+^) are used to best represent the complexes observed. Conditions for **d** (actual injected [Aβ]=10 μM): [Aβ_40_]=100 μM; [Cu(II)]=100 μM; [**4**]=500 μM; 100 mM ammonium acetate (pH 7.5) with 1% v/v DMSO; 37 °C; 1 h incubation.

To investigate the interactions between Aβ and **1**–**4**, nano-electrospray ionization MS (nESI-MS) combined with ion mobility–mass spectrometry (IM–MS), optimized for the detection of non-covalent protein complexes^40^,^41^,^42^, was employed. Data obtained from the samples incubated for 30 min indicated that **1**–**3** exhibited a metal-dependent interaction with both Aβ_40_ and Aβ_42_ (Fig. 5 and Supplementary Fig. 7, respectively). Both **1** and **3** were capable of producing Cu–ligand-dependent signals corresponding to a mass 89 Da lighter than the apo Aβ_40_/Aβ_42_, albeit with clear differences in the abundance of this product. Tandem mass spectrometry (MS^2^) sequencing of the Aβ peak supported that it corresponded to a *N*-terminally truncated form of the peptide (Supplementary Fig. 8 and Supplementary Table 5), consistent with data previously reported for **L2-b** (ref. 25). No data acquired presented a stable interaction between **1** and **3** with either Aβ_40_ or Aβ_42_ under our conditions, indicative of the formation of a transient Cu-containing ternary complex.

**2** produced stable ternary complexes comprising single equivalents of both Aβ_40_/Aβ_42_ and the ligand, with one or two Cu(II) bound. IM–MS data support that binding of **2** to Aβ_40_ shifts the arrival time distributions when compared with the ligand-free complexes (Fig. 5b,c and Supplementary Fig. 7). Because of the increased chemical noise, we were unable to produce IM–MS observations for these compounds in the presence of Aβ_42_. Using the Aβ_40_ as a model system which exhibited reduced chemical noise and kinetics of aggregation, these results demonstrate that the formation of a ternary complex between Cu(II), Aβ and **2** results in an altered downstream Aβ aggregation pathway. This is consistent with previous observations for other small molecules that can modulate metal–Aβ reactivity^25^,^27^. In the absence of Cu(II), the above small molecules were not observed in complex with the peptide (Supplementary Fig. 9).

In addition, the Cu(II)–Aβ_40_/Aβ_42_ samples prepared under the conditions of the inhibition experiments shown in Fig. 2 (for example, buffer, 24 h incubation) were analysed by matrix-assisted laser desorption ionization mass spectrometry (MALDI–MS). As depicted in Supplementary Fig. 10, ions smaller than full-length Aβ_40_ (loss of 89 Da) appeared from the samples treated with **1** and **3**, consistent with the nESI-MS results (vide supra; Fig. 5a). Along with truncation of the peptide, upon incubation of Cu(II)–Aβ with **1** or **3** for 24 h, oxidation of Aβ_40_ was also observed. As indicated by nESI-MS and IM–MS data, Aβ added with **2** was not indicated to be modified even after 24 h (Supplementary Fig. 10). Thus, both events (that is, truncation and oxidation of Aβ) by **1** and **3** with Cu(II) being present might be responsible for redirecting Aβ aggregation pathways.

To determine if intact or transformed **4** would be the active species which could interact with Aβ, both copper-free and copper-present samples incubated with the molecule were also monitored. When **4** was reacted with metal-free Aβ_40_ for 6 h ([Aβ_40_]:[**4**]=1:5, [Aβ_40_]=100 μM), the compound, observed by ESI–MS, was indicated to be degraded showing its multiple fragments and their nonspecific adducts of monomeric and oligomeric Aβ species (Supplementary Fig. 11). Note that in conditions comparable to the nESI-MS studies of **1**–**3** (Fig. 5), **4** was not shown to bind to Aβ_40_ in 30 min and 24 h long incubations at 37 °C (Fig. 5a and Supplementary Fig. 12). In the case of the Aβ samples incubated with **4** and Cu(II) for 1 h, oxidized Aβ was shown, along with formation of covalent complexes of **BQ**^29^, Aβ and/or Cu(II) (Fig. 5d). The generation of oxidized Aβ was also indicated in the sample incubated with Aβ, Cu(II) and **4** for 24 h under the same condition of the studies (Fig. 2), analysed by MALDI–MS (Supplementary Fig. 10).

To identify the oxidation sites in Aβ, MS^2^ analysis was carried out on the non-oxidized and singly oxidized Aβ_40_ (Supplementary Fig. 13). In the MS^2^ of the singly oxidized Aβ, ions larger than *b*_35_ fragments, including the first 35 amino acid residues from the N-terminus, existed as the oxidized form; however, both non-oxidized and oxidized forms were found in fragments smaller than *b*_34_. These results suggest that oxidation can occur at several sites, including the methionine residue (M35; Aβ sequence shown in Fig. 2a). As presented in Supplementary Fig. 13, the smallest oxidized ion is *b*_13_ and no oxidation was observed in the *b*_9_ fragment. Thus, along with M35 (ref. 43), H13 and H14 could be additional plausible oxidation sites in Aβ, as previously reported^44^,^45^.

Together, our overall MS studies suggest that **1**–**4** can interact with metal-free Aβ and/or Cu(II)-bound Aβ in different manners. The distinct redirecting activity of **1**–**4** towards metal-free and metal-treated Aβ aggregation pathways, indicated in both inhibition and disaggregation experiments (vide supra), could be directed by multiple mechanisms, including Aβ modifications (that is, degradation and oxidation), non-covalent complex formation, and covalently linked adduct generation. The overall proposed mechanisms of **1**–**4** towards individual targets are described in detail in the following sections (vide infra).

### Computational studies for prediction of hydrolysis

**Figure 6:**
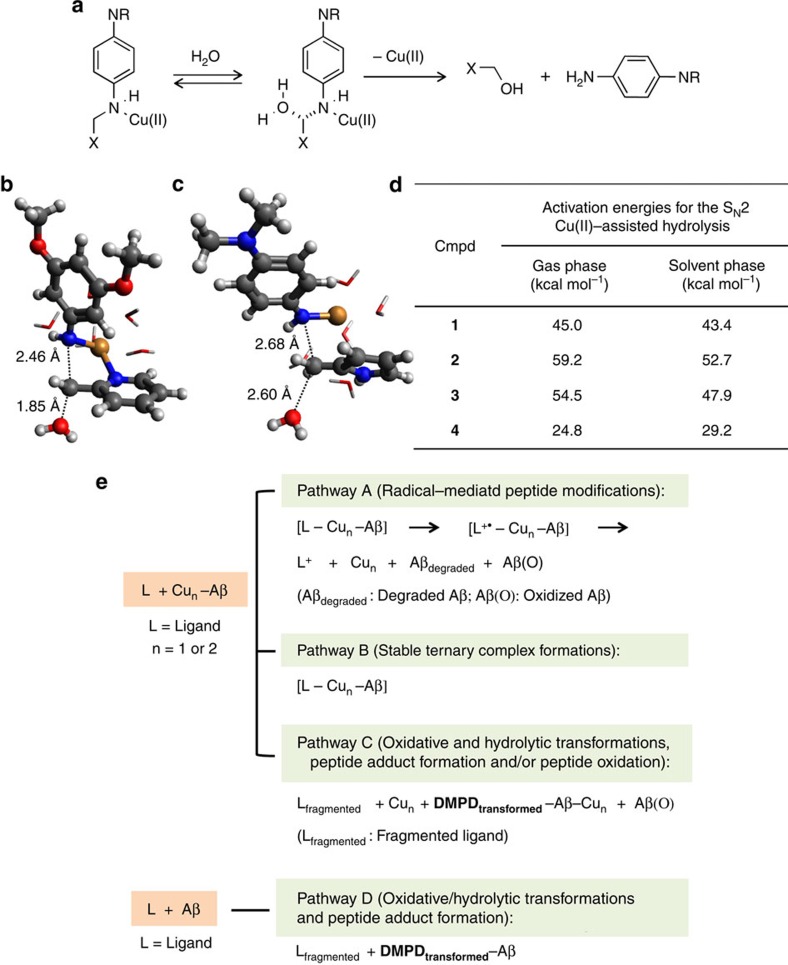
Computational investigations of the S_N_2 Cu(II)-assisted hydrolysis and proposed mechanisms. (**a**) Scheme of the model used to calculate the S_N_2 Cu(II)-assisted hydrolysis of **1**–**4**. (**b**,**c**) The calculated transition state structures of the hydrolysis of (**b**) **2** and (**c**) **4**. The bond lengths of bonds being broken and being formed are shown. For clarity, the water molecules coordinated to Cu(II) are represented with sticks (red: O; blue: N; grey: C; white: H; orange: Cu). (**d**) Calculated activation energies for the S_N_2 Cu(II)-assisted hydrolysis in the gas and solvent (water) phases for **1**–**4**. (**e**) Proposed mechanisms for the different activities of **1**–**4** towards metal-free or metal-associated Aβ species under aerobic conditions.

Along with calculations of IP_1_ and IP_2_ values (Fig. 1b–d, vide supra), to further elucidate the modes of action of **1**–**4** towards Aβ in the presence of Cu(II), density functional theory calculations were employed (Fig. 6a–d). A simplified model calculation on the S_N_2-type cleavage of the C–N bond showed that **4** had the lowest barrier for hydrolysis resulting in the production of **DMPD** (Fig. 6c,d). This is consistent with the UV–vis and MS studies (vide supra). Thus, **4** possibly reacts with both metal-free Aβ and metal–Aβ, as indicated by **DMPD**^29^.

### Proposed mechanisms for reactivities of 1–4 with targets

Collecting the overall results from our optical, ESI–MS/IM–MS/MALDI–MS, and computational investigations, we propose the multiple modes of action of **1**–**4** towards targets. First, the modes of action of our compounds to mediate the aggregation of metal-free Aβ and metal-Aβ are suggested (Fig. 6e). Studies presented herein indicate that the mode of action of **1** and **3** could involve the formation of a transient ternary complex between Aβ, Cu(II) and compound. This could be subsequently followed by the oxidation of **1** and **3** as well as the oxidation and/or degradation of the peptide by well-documented radical pathways (proposed pathway A: radical-mediated peptide modifications; Fig. 6e)^43^,^44^,^45^,^46^,^47^,^48^. **2** is most likely to undergo a different pathway of Aβ aggregation modulation due to its higher IPs (Fig. 1d). Thus, it is proposed that the interaction of **2** with Cu(II)–Aβ results in the formation of a stable ternary complex consisting of Aβ, Cu(II) and **2** which subsequently diverts Cu(II)–Aβ aggregation pathways producing off-pathway species with different conformations (proposed pathway B; stable ternary complex formation; Fig. 6e). In the case of **4**, based on the density functional theory calculations, along with the experimentally observed Aβ–**DMPD_transformed_** adducts, this molecule might be transformed to **DMPD** *via* oxidative and hydrolytic pathways^29^, which could be responsible for interaction and reactivity with both metal-free and metal-associated Aβ through the proposed modes of action C and D (oxidative/hydrolytic transformations, peptide adduct formation and/or peptide oxidation; Fig. 6e).

In addition, **1**–**4** are able to mediate oxidative stress caused by the presence of free organic radicals by acting as antioxidants to donate an electron to quench the radials^49^. This is evidenced by their activity towards the radicals roughly correlating with the calculated IPs (Fig. 1d) which measure the ease of releasing an electron. Furthermore, in the presence of Cu(I/II), **1**–**3** can also inhibit ROS generation. This activity is possibly originated by **1**–**3** binding to copper and preventing it being reduced to form ROS (for example, hydroxyl radicals)^49^, along with compounds' antioxidant activity. The previously discussed electronic properties (for example, IPs, Fig. 1d) of the compounds could also compliment this activity. More detailed investigations of the modes of action of **1**–**4** towards targets will be the subject of future studies.

## Discussion

To date, effective drugs against AD are not available since the pathological factors and pathways of AD are still unclear. To identify such pathological features associated with AD, chemical tools with distinct specificity towards various individual or associated targets are needed. Herein, we designed four small molecules based on a structure-mechanism-based design strategy for targeting and regulating distinct pathological factors (for example, metal ions, metal-free Aβ, metal–Aβ, ROS, free organic radicals) linked to AD pathology as chemical tools useful for AD research. Our studies indicate that the desired chemical properties of small molecules can be achieved for reactions with pathological features simply through minor structural variations to a parent framework. In addition, such property tuning of small molecules is observed to successfully afford different modes of action towards the targets. Furthermore, along with *in vitro* characterizations at the molecular level, the validation of our chemical tool in the 5 × FAD AD mouse model confirms its utility in investigating AD aetiology. Therefore, our findings of the small molecules, able to probe distinct pathological facets *via* disparate mechanisms, demonstrate the feasibility of applying a structure-mechanism-based design concept to rationally construct chemical tools capable of illuminating the roles of multiple individual targets and their inter-relationships in AD pathogenesis. Moreover, such chemical tools will provide an unconventional avenue for discovering effective diagnostics and therapeutics for AD.

## Methods

### Parallel artificial membrane permeability assay adapted for BBB (PAMPA-BBB)

PAMPA-BBB experiments were conducted using the PAMPA Explorer kit (*p*ION Inc., Billerica, MA, USA) using previously reported protocols^24^,^28^,^29^. Compounds (25 μM, 200 μl) in pH 7.4 Prisma HT buffer (*p*ION) were added to the wells of a donor plate (number of replicates=12). The polyvinylidene fluoride (PVDF, 0.45 μM) filter membrane on the acceptor plate was coated with BBB-1 lipid formulation (5 μl, *p*ION). The acceptor plate was placed on top of the donor plate. Brain sink buffer (BSB, 200 μl, *p*ION) was added to each well of the acceptor plate and was incubated for 4 h at ambient temperature without stirring. UV–vis spectra of the solutions in the reference, acceptor, and donor plates were measured using a microplate reader. The PAMPA Explorer software v. 3.5 (*p*ION) was used to calculate the −log*P*_e_ values for compounds. CNS±designations were assigned by comparison with compounds that were identified in previous reports^50^,^51^,^52^.

### Aβ aggregation experiments

Before experiments, Aβ_40_ or Aβ_42_ was dissolved in ammonium hydroxide (NH_4_OH; 1% v/v, aq). The resulting solution was aliquoted, lyophilized overnight and stored at −80 °C. A stock solution of Aβ was then prepared by dissolving lyophilized peptide in 1% NH_4_OH (10 μl) and diluting with ddH_2_O. The concentration of the solution was determined by measuring the absorbance of the solution at 280 nm (*ɛ*=1,450 M^–1^ cm^–1^ for Aβ_40_; *ɛ*=1,490 M^–1^ cm^–1^ for Aβ_42_). The peptide stock solution was diluted to a final concentration of 25 μM in Chelex-treated buffered solution containing HEPES (20 μM; pH 6.6 for Cu(II) samples; pH 7.4 for metal-free and Zn(II) samples) and NaCl (150 μM). For the inhibition studies, compounds (final concentration 50 μM, 1% v/v dimethyl sulfoxide (DMSO)) were added to the sample of Aβ (25 μM) in the absence and presence of a metal chloride salt (CuCl_2_ or ZnCl_2_; 25, 50, 100 or 125 μM) followed by incubation at 37 °C with constant agitation for 24 h. For the disaggregation studies, Aβ with and without a metal chloride salt was incubated for 24 h at 37 °C with constant agitation to generate preformed Aβ aggregates. The resulting samples were then treated with compounds (50 μM) and incubated with constant agitation for additional 24 h.

### Gel electrophoresis and western blotting

The Aβ samples from *in vivo* or *in vitro* experiments were analysed by gel electrophoresis followed by western blotting using an anti-Aβ antibody (6E10)^24^,^25^,^27^,^28^,^29^. Samples (10 μl) were separated on a 10–20% Tris-tricine gel (Invitrogen, Grand Island, NY, USA). Following separation, the proteins were transferred onto nitrocellulose membranes and blocked with bovine serum albumin (BSA, 3% w/v, Sigma-Aldrich, St Louis, MO, USA) in Tris-buffered saline (TBS) containing 0.1% Tween-20 (TBS-T) for 2 h at room temperature or overnight at 4 °C. The membranes were incubated with an anti-Aβ antibody (6E10, 1:2,000, Covance, Princeton, NJ, USA) in a solution of 2% BSA (w/v in TBS-T) for 4 h at room temperature or overnight at 4 °C. After washing with TBS-T (3 × , 10 min), a horseradish peroxidase-conjugated goat anti-mouse secondary antibody (1:5,000 in 2% BSA w/v in TBS-T; Cayman Chemical Company, Ann Arbor, MI, USA) was added for 1 h at room temperature. The Thermo Scientific SuperSignal West Pico Chemiluminescent Substrate (Thermo Scientific, Rockford, IL, USA), Biosesang ECL Plus kit (Biosesang, Gyeonggi-do, Republic of Korea), or a homemade ECL kit^53^ was used to visualize the results on a ChemiDoc MP Imaging System (Bio-Rad, Hercules, CA, USA).

### Transmission electron microscopy (TEM)

Samples for TEM were prepared according to a previously reported method using glow-discharged grids (Formar/Carbon 300-mesh, Electron Microscopy Sciences, Hatfield, PA, USA)^24^,^25^,^27^,^28^,^29^. Images for each sample were taken on a JEOL JEM-2100 transmission electron microscope (UNIST Central Research Facilities, Ulsan National Institute of Science and Technology, Ulsan, Republic of Korea).

### Cell viability studies

The M17 cell line was purchased from the American Type Culture Collection (ATCC, Manassas, VA, USA). The cell line was maintained in media containing 50% minimum essential medium and 50% F12 (GIBCO, Grand Island, NY, USA), supplemented with 10% fetal bovine serum (Sigma), 100 U ml^−1^ penicillin, and 100 mg ml^−1^ streptomycin (GIBCO). The cells were grown and maintained at 37 °C in a humidified atmosphere with 5% CO_2_. The cell culture used in this work did not indicate mycoplasma contamination. Cell viability upon treatment of compounds was determined using the MTT assay (Sigma). M17 cells were seeded in a 96-well plate (15,000 cells in 100 μl per well). The cells were treated with Aβ (20 μM) with or without CuCl_2_ or ZnCl_2_ (20 μM), followed by the addition of compounds (20 μM, 1% v/v final DMSO concentration), and incubated for 24 h. After incubation, 25 μl MTT (5 mg ml^−1^ in phosphate buffered saline (PBS, pH 7.4, GIBCO) was added to each well and the plate was incubated for 4 h at 37 °C. Formazan produced by the cells was solubilized using an acidic solution of *N*,*N*-dimethylformamide (DMF, 50%, v/v aq) and SDS (20%, w/v) overnight at room temperature in the dark. The absorbance was measured at 600 nm using a microplate reader. Cell viability was calculated relative to cells containing an equivalent amount of DMSO.

### TEAC assay

The assay employing M17 cell lysates was conducted following the protocol of the antioxidant assay kit purchased from Cayman Chemical Company (Ann Arbor, MI, USA) with minor modifications^25^,^28^,^29^. For the antioxidant assay using cell lysates, cells were seeded in a six-well plate and grown to ∼80–90% confluence. Cell lysates were prepared following a previously reported method with modifications^54^. M17 cells were washed once with cold PBS (pH 7.4, GIBCO) and harvested by gently pipetting off adherent cells with cold PBS. The cell pellet was generated by centrifugation (2,000*g* for 10 min at 4 °C). This cell pellet was sonicated on ice (5 s pulses, three times with 20 s intervals between each pulse) in 2 ml of cold Assay Buffer (5 mM potassium phosphate, pH 7.4, containing 0.9% NaCl and 0.1% glucose). The cell lysates were centrifuged at 5,000*g* for 10 min at 4 °C. The supernatant was removed and stored on ice until use. For standard and samples in 96-well plates, 10 μl of the supernatant of cell lysates was delivered followed by addition of compound, metmyoglobin, ABTS and H_2_O_2_ in order. After 5 min incubation at room temperature on a shaker, absorbance values at 750 nm were recorded. The antioxidant concentration was calculated according to the measured absorbance (% inhibition=100 × (A_0_ – A)/A_0_, where A_0_ is absorbance of the supernatant of cell lysates). The measurements were conducted in triplicate.

### 2-Deoxyribose assay

The ability of **1**–**3** to decrease free radical formation from Fenton-like chemistry by Cu(I/II) was determined using previously reported procedures^28^,^30^. To summarize, solutions of phosphate buffer (50 mM NaH_2_PO_4_, pH 7.2) treated with Chelex overnight, compound (125 μM in water), CuCl_2_ (10 μM), 2-deoxy-D-ribose (15 mM), H_2_O_2_ (200 μM), and sodium ascorbate (2 mM) were mixed in the listed order and incubated at 37 °C with constant agitation. These conditions were chosen to optimize the formation of the chromogen produced during the course of the assay. After 1 h, the samples were quenched with trichloroacetic acid (2.8% w/v, 200 μl) and 2-thiobarbituric acid (1% w/v, 200 μl) and heated at 100 °C for 20 min. The samples were allowed to cool for 5 min before measuring the absorbance values at 532 nm on a microplate reader. Samples without compounds were also tested as a control. Normalized absorbance values were calculated as previously reported^28^.

### Determination of solution speciation for 2 and Cu(II)–2 complex

The p*K*_a_ value for **2** was determined by UV–vis variable-pH titrations as previously reported^24^,^55^. To establish the p*K*_a_ value, a solution (10 mM NaOH, pH 12, 100 mM NaCl) of **2** (100 μM) was titrated with small amounts of HCl. At least 30 spectra were recorded in the range of pH 2–10. Similarly, a solution containing CuCl_2_ and **2** (50 μM) in a metal to ligand ratio of 1:2 was titrated with small additions of HCl and at least 30 spectra were recorded over the range pH 2–7. The acidity and stability constants were calculated by using the HypSpec program (Protonic Software, UK)^56^. Speciation diagrams were modelled in the HySS2009 program (Protonic Software)^57^.

### Stability studies

The stability of **1**–**4** (50 μM) in the absence and presence of CuCl_2_ (25 μM) was monitored every 10 min using UV–vis for 5 h in buffer (20 μM HEPES, pH 7.4, 150 μM NaCl; 1% DMSO) at 37 °C. The resulting spectra were corrected for baseline shifts at 800 nm and the half-life and rate of decay of the absorbance at 250, 385 and 400 nm for **4**, [Cu(II)+**1**], and [Cu(II)+**3**], respectively, was calculated using the first-order exponential decay function as implemented in Origin 9.1 (OrginLab Corp., Northampton, MA, USA). Additionally, the species present were identified using ESI–MS. Samples containing **1**–**4** (50 μM) with or without CuCl_2_ (25 μM) were incubated in ddH_2_O (1% DMSO) at 37 °C for the selected time points before being freshly frozen using liquid nitrogen and stored at −80 °C until they were thawed immediately before measurement.

### Ion mobility–mass spectrometry (IM–MS)

All IM–MS experiments were carried out on a Synapt G2 (Waters, Milford, MA)^27^,^28^,^29^,^58^. Samples were ionized using a nano-electrospray source operated in positive ion mode. MS instrumentation was operated at a backing pressure of 2.7 mbar and sample cone voltage of 40 V. The *m*/*z* scale was calibrated using 20 mg ml^−1^ aqueous cesium iodide. For peptide-metal ligation studies, the aliquots of Aβ peptides (final concentration 18 μM) were sonicated for 5 s before pre-incubation with or without a source of Cu(II) (copper(II) acetate) at 37 °C for 10 min. After pre-incubation, the samples were treated with or without **1**–**4** (final concentration 160 μM) and incubated at 37 °C for 30 min before analysis. Solution conditions were 100 mM ammonium acetate (pH 7.5) with 1% v/v DMSO. Covalent binding studies with **4** were performed by incubating aliquots of Aβ with the ligand at a ratio of 1:0, 1:5 and 1:25 in water for either 24 h or a week at 25 °C. After incubation the samples were lyophilized for storage until analysis. Immediately before analysis samples were reconstituted to 50 μM peptide concentration in 1,1,1,3,3,3-hexafluoro-2-propanol, sonicated and diluted further to 25 μM for data acquisition. Accurate mass values for ligand-bound complexes were calculated using the monoisotopic peak difference between apo and ligated states with errors reported as a function of two times the s.d. collision cross section (CCS) measurements were externally calibrated using a database of known values in helium, using values for proteins that bracket the likely CCS and ion mobility values of the unknown ions^58^,^59^. CCS values are the average of six replicates with errors reported as the least square product. This least square analysis combines inherent calibrant error from drift tube measurements (3%)^59^. calibration curve error, and two times the replicate s.d. error. All other conditions are consistent with previously published methods^25^.

### Electrospray ionization mass spectrometry (ESI–MS)

Aβ (100 μM) was incubated with **4** (500 μM) in 100 mM ammonium acetate, pH 7.5, without and with the addition of CuCl_2_ (100 μM) for 1 h at 37 °C without agitation. Before injection into the mass spectrometer, the resulting Aβ was diluted by 10-fold. ESI–MS analysis was performed using a Synapt G2-Si quadrupole time-of-flight mass spectrometer (Waters, Manchester, UK) equipped with electrospray ionization source. The capillary voltage, sampling cone voltage, and source temperature were set to 2.8 kV, 70 V, and 60 °C, respectively. The backing pressure was adjusted to 3.2 mbar.

### Matrix-assisted laser desorption ionization mass spectrometry (MALDI–MS)

Aβ samples (prepared in the same procedure with Aβ aggregation experiments) were mixed with the equivalent volume of the matrix solution and loaded on the target plate. The matrix solution was prepared with α-cyano-4-hydroxycinnamic acid (Sigma-Aldrich) by dissolving in 40% CH_3_CN and 2% trifluoroacetic acid and adjusting the concentration to 5 mg ml^−1^. MALDI–MS analysis was conducted using an Ultraflex III time-of-flight mass spectrometer (Bruker Daltonics, Bremen, Germany). Mass spectra were acquired over the range of 1,000–6,000 *m*/*z*.

### Calculation of transition state energies and ionization potentials

First-principles calculations using Gaussian09 (ref. 60) were carried out for **1**–**4** in order to study the S_N_2 hydrolysis and their one and two-electron oxidation. For the direct C–N bond hydrolysis mechanism assisted by Cu(II), five additional water molecules were added to the first hydration sphere of Cu(II) in addition to the water molecule acting as a nucleophile. The structure optimizations were performed in the vacuum at M06/6-31G(d) level. The Los Alamos effective-core potential (ECP) LanL2DZ basis set was applied for Cu(II). The hydration effect was taken into account by additional single point calculations with polarizable continuum model at M06/6-31G(d) level. To find transition states as the first-order saddle points on the PES, Berny algorithm was used. The validity of the transition states were confirmed by frequency calculations (one imaginary) corresponding to the translational motion of the carbon from C–N bond. For the two-electron oxidation of **1**–**4**, we calculated two successive adiabatic ionization potentials accounting for the sequential loss of electrons. The radical cation from the first ionization is assumed to undergo immediate deprotonation before the second ionization. All the relevant chemical species were optimized at M06/6-31G(d) level, and their thermodynamic parameters were calculated at M06/6-311+G(2df,2p) level. Inclusion of the solvation effect of water using the polarizable continuum model reduces the ionization potential, but the overall trend is conserved.

### Animal studies

The amyloidogenic characteristics of 5 × FAD mice have been described previously^29^. In brief, 5 × FAD mice express the Swedish/London/Florida mutations of the human amyloid precursor protein (hAPP) and the M146L/L286V mutations of the human presenilin-1 (PSEN1). They develop the early and robust pathology of AD with the cognitive and behavioural impairments. The 5 × FAD mice have been widely used to test the possible efficacy of a compound targeted for AD^25^,^29^. We maintained the mice on a C57BL/6 × SJL hybrid background. All animal experiments were performed in accordance with the guidelines of the Asan Institute for Life Science for Laboratory Animal Care and Use (Seoul, Korea), where the animals had free access to water and food, and were housed on 12 h light/12 h dark cycle.

### Compound treatment to animals

Chemicals used were freshly prepared shortly before administration to the animals. We treated 5 × FAD mice with **1** (1 mg kg^−1^ of body weight) or the vehicle (1% v/v DMSO in 20 mM HEPES, pH 7.4, 150 mM NaCl) starting at 3 months of age, which is the same method used in our previous studies^25^,^29^. **1** was daily injected into the lower abdomen of the 5 × FAD mice for 30 days.

### Assessment of cognitive function by the Morris water maze test

Mice were subjected to the Morris water maze test to assess their performance of spatial learning and memory^25^,^61^. The water maze was a circular plastic pool (120 cm diameter) and filled with murky water (21.0±1.0 °C). A cylindrical escape platform (15 cm diameter) stood 0.5 cm under the water surface. Three hours after the 30th compound treatment, the mice were allowed the first training to swim and find the hidden escape platform in the water with three repeats per training. Thereafter, they daily tried the task on the next 4 consecutive days. Three hours after the final escape test, the platform was removed, and the mice exercised a probe trial for 60 s. The animal performance was traced with SMART Video Tracking System (Harvard Apparatus, Holliston, MA, USA).

### Tissue preparation

Immediately after the behavioural test, the mice were sacrificed under deep anaesthesia. The brain was divided into left and right cerebral hemispheres for biochemical and histological analysis, respectively and quickly frozen in liquid nitrogen.

### ELISA for quantification of the cerebral Aβ

We measured the levels of various types of Aβ in the brain as described previously^25^,^29^. The protein fractions were serially prepared from the left hemispheres in PBS (pH 7.4), in 2% SDS and in 70% FA, and then subjected to the human Aβ_40_ and Aβ_42_ ELISA quantification according to the manufacturer's methods (Invitrogen, Carlsbad, CA, USA). The aggregated (Invitorgen) and oligomeric Aβ (82E1-specific; IBL International, Hamburg, Germany) ELISA quantifications were also performed using PBS fractions. The cerebral levels of Aβ_40_ and Aβ_42_ were represented as moles per gram of wet brain tissue, whereas the aggregated or oligomeric Aβ amounts were expressed as grams per gram of wet brain tissue.

### Histological quantification of cerebral amyloid pathology

12-μm sagittal sections of the brain were prepared from the right hemispheres on a cryostat (HM550; Microm, Walldorf, Germany), and mounted onto 1% poly-L-lysine-coated glass slides. Immunohistochemistry was performed on the tissue section using the human Aβ(17–24)-specific antibody 4G8 (Covance, Princeton, NJ, USA). They were immunologically reacted with 4G8 (1:1,000 dilution) and biotinylated anti-mouse IgG secondary antibody (Vector Laboratories, Burlingame, CA, USA), and then developed with 0.015% diaminobenzidine/0.001% H_2_O_2_ (in PBS; Vector Laboratories). The immuno-reacted sections were examined or photographed under a light microscope (Eclipse 80i; Nikon, Tokyo, Japan). The congophillic amyloid plaques were examined after staining the sections with Accustain Congo Red amyloid staining solution (Sigma). The loads of amyloid deposits in the brain were expressed as the per cent area of 4G8-immunoreactive deposits or the number of congophilic plaques per mm^2^ of a cortical region of interest.

### Statistics

Data are expressed as mean±s.e.m. Statistical differences between groups were determined with the unpaired *t*-test. Statistical significance was considered at *P*<0.05. The current animal study was performed in parallel with the study previously reported^29^ using the same control groups (vehicle-treated wild-type and 5 × FAD mice); thus, the same control data were used in both studies.

### Data availability

All relevant data are available from the authors upon request.

## Additional information

**How to cite this article:** Beck, M. W. *et al*. Structure-mechanism-based engineering of chemical regulators targeting distinct pathological factors in Alzheimer's disease. *Nat. Commun.* **7,** 13115 doi: 10.1038/ncomms13115 (2016).

## Supplementary Material

Supplementary Information Supplementary Figures 1 - 13, Supplementary Tables 1 - 5, Supplementary Methods and Supplementary References
