# Supplementary material for: Structure-mechanism-based engineering of chemical regulators targeting distinct pathological factors in Alzheimer's disease
Source: Nat Commun. 2016 Oct 13;7:13115. doi: 10.1038/ncomms13115 (PMC5065625; doi:10.1038/ncomms13115)
Supplement: Supplementary Information — Supplementary Figures 1 - 13, Supplementary Tables 1 - 5, Supplementary Methods and Supplementary References [file ncomms13115-s1.pdf]

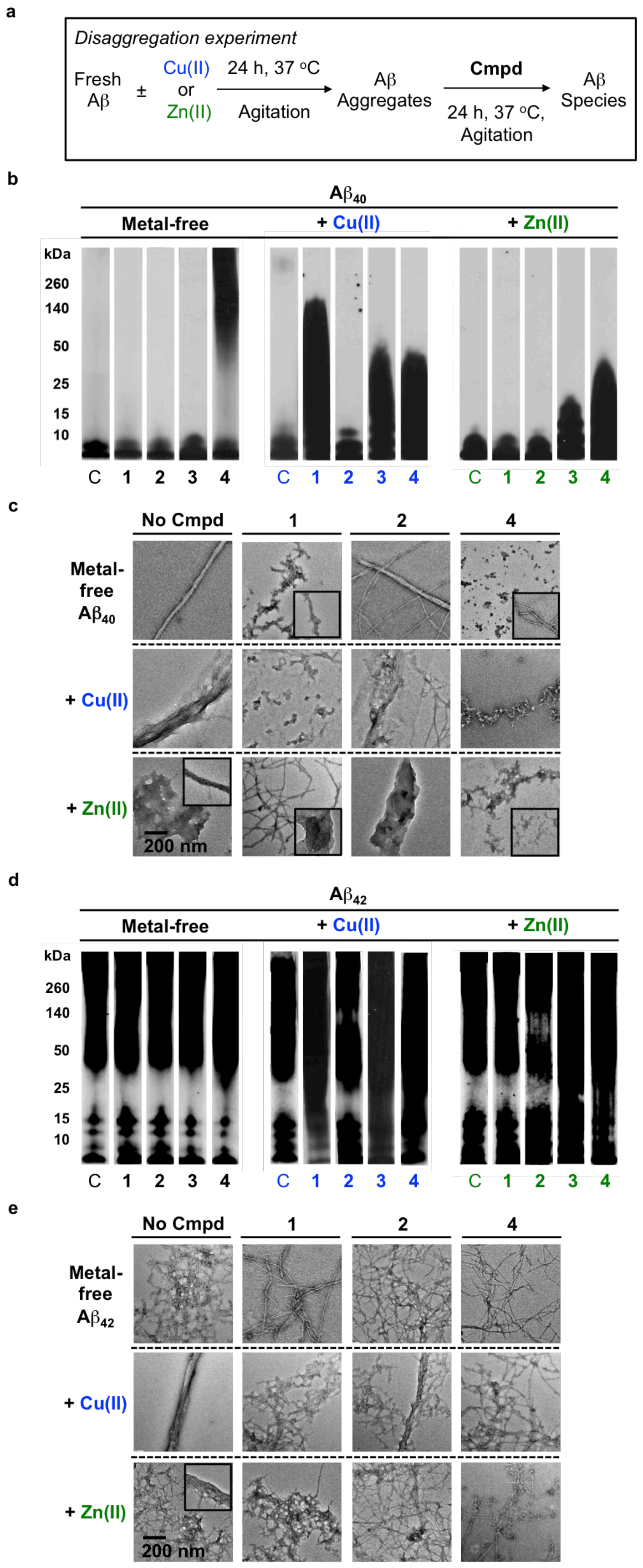
a
Disaggregation experiment
Fresh Aβ
±
Cu(II) or Zn(II)
24 h, 37 °C
Agitation
Aβ Aggregates
Cmpd
24 h, 37 °C, Agitation
Aβ Species
b
Aβ40
Metal-free
+ Cu(II)
+ Zn(II)
kDa
260
140
50
25
15
10
C 1 2 3 4
C 1 2 3 4
C 1 2 3 4
c
No Cmpd
1
2
4
Metal-free Aβ40
+ Cu(II)
+ Zn(II)
200 nm
d
Aβ42
Metal-free
+ Cu(II)
+ Zn(II)
kDa
260
140
50
25
15
10
C 1 2 3 4
C 1 2 3 4
C 1 2 3 4
e
No Cmpd
1
2
4
Metal-free Aβ42
+ Cu(II)
+ Zn(II)
200 nm

**Supplementary Figure 1 | Ability of 1-4 to disrupt preformed metal-free and metal−Aβ aggregates.** (**a**) Scheme of the disaggregation experiment: Metal-free and metal treated Aβ aggregates were generated by incubating mixtures of freshly prepared $A\beta_{40}$ or $A\beta_{42}$ (25 μM) in the presence or absence of Cu(II) (blue, 25 μM) or Zn(II) (green, 25 μM) at 37 °C with agitation. After 24 h, the samples were treated with **1-4** (50 μM) and incubated for an additional 24 h. Gel electrophoresis and Western blot analysis of the molecular weight distribution of the resulting (**b**) $A\beta_{40}$ and (**d**) $A\beta_{42}$ species using an anti-Aβ antibody (6E10). Morphologies of the (**c**) $A\beta_{40}$ and (**e**) $A\beta_{42}$ species as observed using TEM (scale bar = 200 nm).

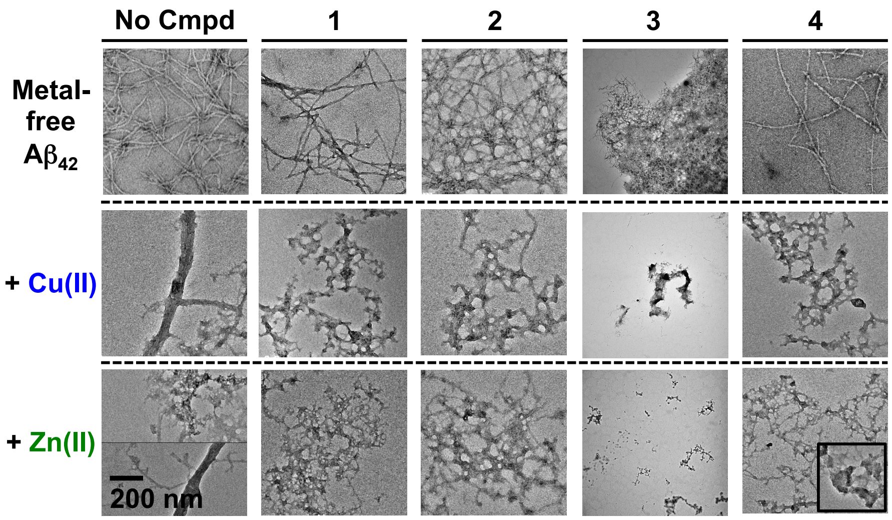


**Supplementary Figure 2 | Ability of 1-4 to change the morphology of the resultant $A\beta_{42}$ species from the inhibition experiment** (**Fig. 2**). Images were obtained by TEM (scale bar = 200 nm).

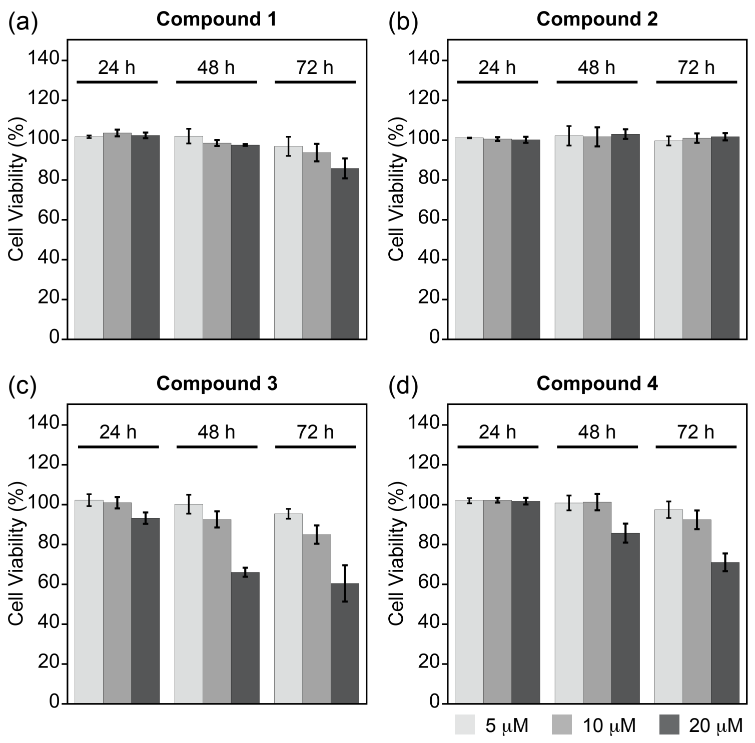


**Supplementary Figure 3 | Toxicity of 1-4 at different concentrations (5 to 20 μM) and incubation time points (24 to 72 h) in M17 cells.** Viability of cells (%) was calculated relative to that of cells incubated only with 1% v/v DMSO. Error bars represent the standard deviation (s.d.) from three independent experiments ($P < 0.05$).

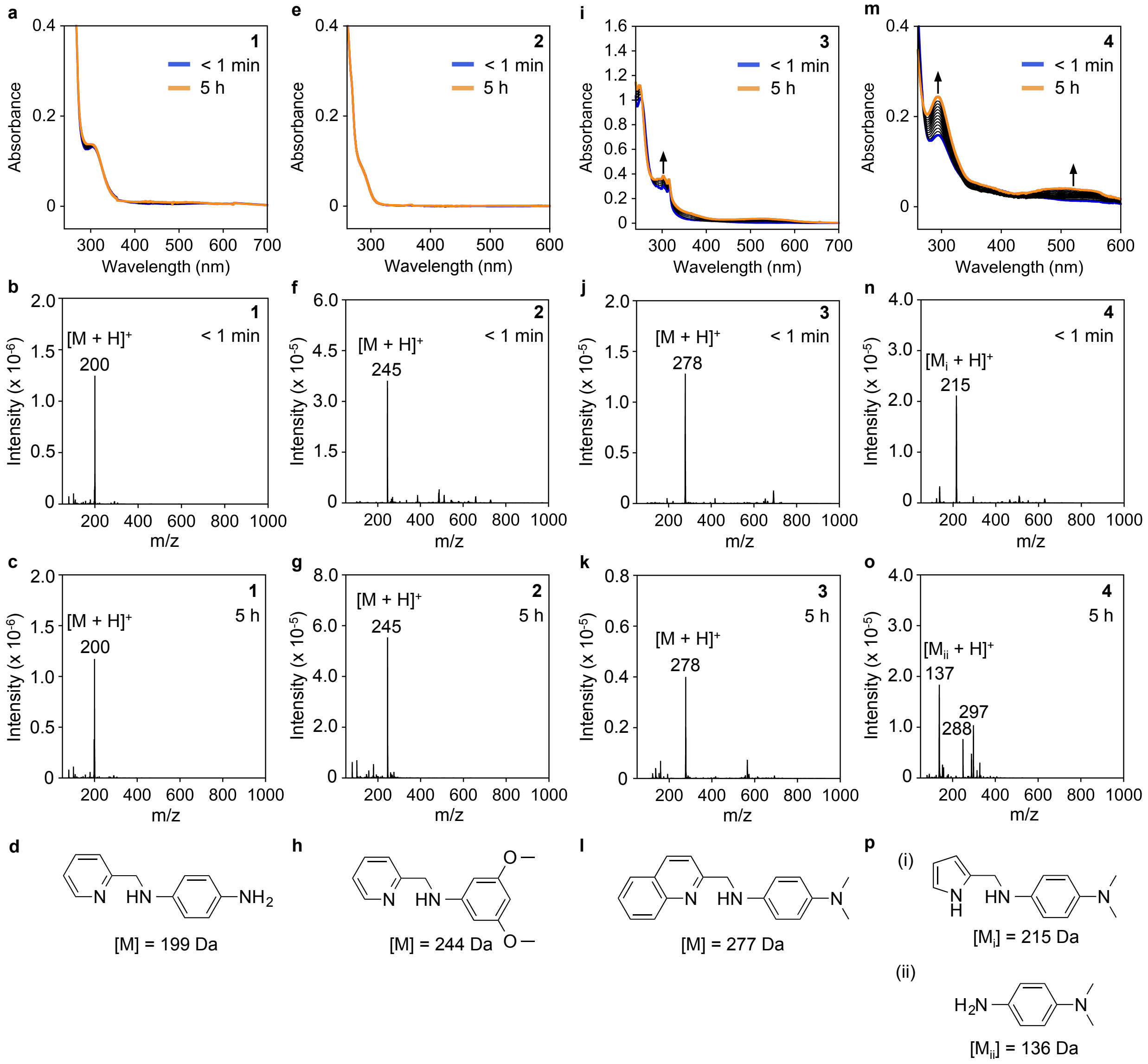


**Supplementary Figure 4 | Stability studies of 1-4 in metal-free conditions by UV–Vis and ESI–MS.** The stability of **1-4** (50 μM) by (**a**,**e**,**i**,**m**) UV-Vis in 20 μM HEPES, pH 7.4, 150 μM NaCl over 5 h (blue: immediately after addition of the sample; orange: after 5 h incubation at 37 $^{o}$C) and by ESI–MS (**b**,**f**,**j**,**n**) immediately after addition of the sample and (**c**,**g**,**k**,**o**) after incubation at 37 $^{o}$C for 5 h in dd$H_2$O. (**d**,**h**,**l**,**p**) Structures and masses of molecules observed in the ESI-MS studies.

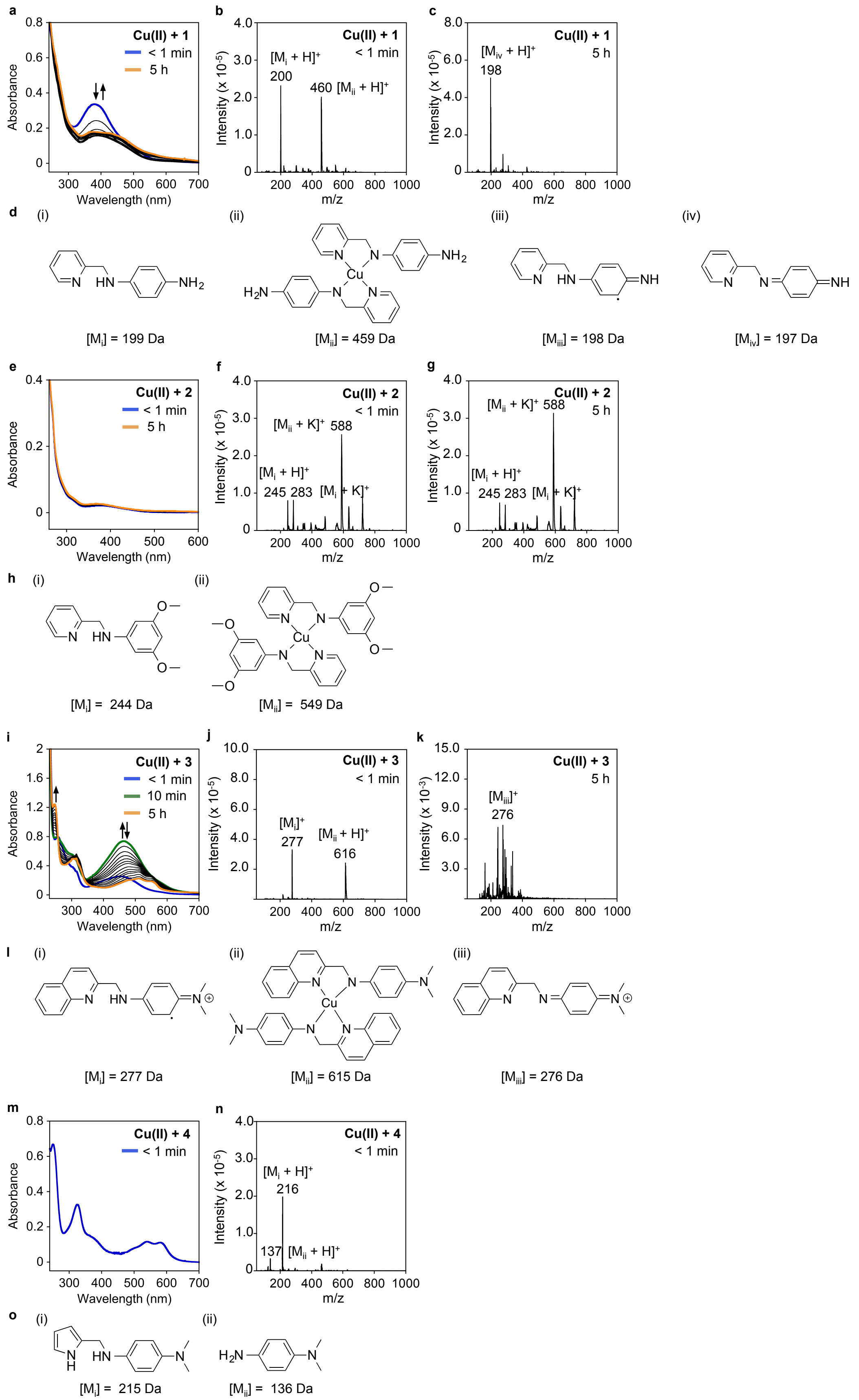
a
Cu(II) + 1
< 1 min
5 h
Absorbance
Wavelength (nm)
b
Cu(II) + 1
< 1 min
[Mi + H]+
200
460 [Mii + H]+
Intensity (x 10-5)
m/z
c
Cu(II) + 1
5 h
[Miv + H]+
198
Intensity (x 10-5)
m/z
d
(i)
(ii)
(iii)
(iv)
[Mi] = 199 Da
[Mii] = 459 Da
[Miii] = 198 Da
[Miv] = 197 Da
e
Cu(II) + 2
< 1 min
5 h
Absorbance
Wavelength (nm)
f
Cu(II) + 2
< 1 min
[Mii + K]+ 588
[Mi + H]+
245 283
[Mi + K]+
Intensity (x 10-5)
m/z
g
Cu(II) + 2
5 h
[Mii + K]+ 588
[Mi + H]+
245 283
[Mi + K]+
Intensity (x 10-5)
m/z
h
(i)
(ii)
[Mi] = 244 Da
[Mii] = 549 Da
i
Cu(II) + 3
< 1 min
10 min
5 h
Absorbance
Wavelength (nm)
j
Cu(II) + 3
< 1 min
[Mi]+
277
[Mii + H]+
616
Intensity (x 10-5)
m/z
k
Cu(II) + 3
5 h
[Miii]+
276
Intensity (x 10-3)
m/z
l
(i)
(ii)
(iii)
[Mi] = 277 Da
[Mii] = 615 Da
[Miii] = 276 Da
m
Cu(II) + 4
< 1 min
Absorbance
Wavelength (nm)
n
Cu(II) + 4
< 1 min
[Mi + H]+
216
137
[Mii + H]+
Intensity (x 10-5)
m/z
o
(i)
(ii)
[Mi] = 215 Da
[Mii] = 136 Da

**Supplementary Figure 5 | Stability studies of 1-4 in the presence of Cu(II) by UV–Vis and ESI–MS.** The stability of **1-4** (50 μM) in the presence of Cu(II) (25 μM) by (**a**,**e**,**i**,**m**) UV-Vis in 20 μM HEPES, pH 7.4, 150 μM NaCl over 5 h (blue: immediately after addition of the sample; green: after 10 min incubation; orange: after 5 h incubation at 37 °C) and by ESI–MS (**b**,**f**,**j**,**n**) immediately after addition of the sample and (**c**,**g**,**k**) after incubation at 37 °C for 5 h in dd$H_2O$. (**d**,**h**,**l**,**o**) Structures and masses of molecules observed in the ESI-MS studies.

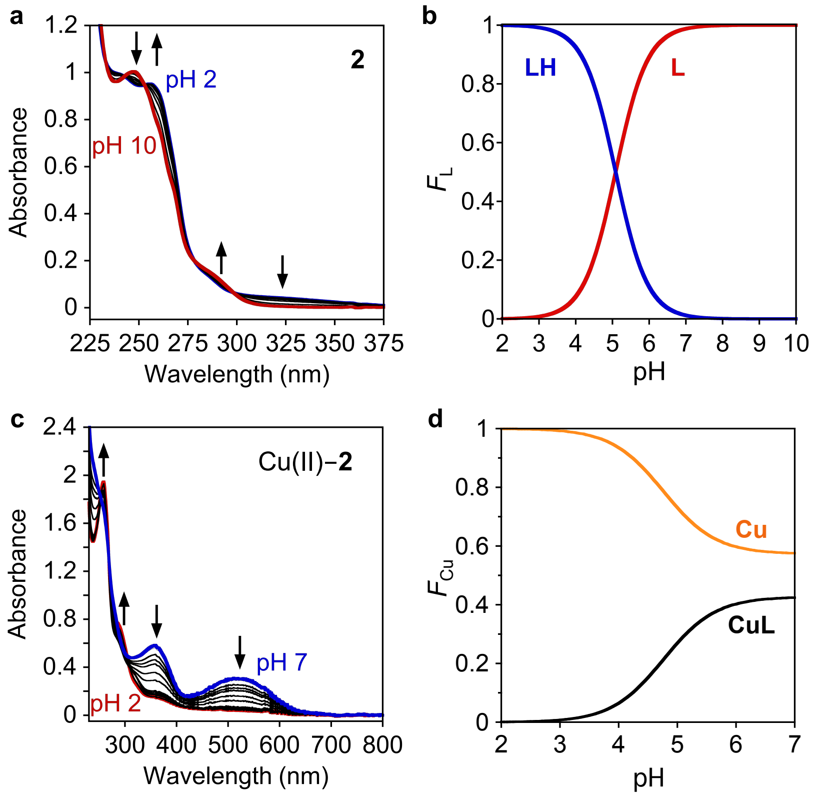


**e**

| | logβ |
|---|---|
| L = | **2** |
| **Cu** + **L** ⇌ **CuL** | 5.6(8) |
| pCu (=−log[Cu(II)]$_{free}$; pH 6.6) | 6.24 |

**Supplementary Figure 6 | Solution speciation studies of 2 and Cu(II)–2 complexes.** (**a**) Variable-pH UV–Vis titration spectra for **2** (100 μM). The resulting spectra were fitted to obtain the p$K_a$ value [p$K_a$ = 5.0(8)] and the speciation diagram (**b**). $F_L$ = Fraction of ligand with at the specified protonation state. (**c**) Variable-pH UV–Vis titration spectra for Cu(II)–**2**. (**d**,**e**) The speciation diagram ($F_{Cu}$ = Fraction of free Cu and Cu(II)–L) and the stability constants (log$\beta$) of Cu(II)–**2**. The parenthesis indicates the error in the last digit of the values. Conditions: Cu(II):L = 1:2, [**2**] = 50 μM; samples were incubated at room temperature for 24 h before titrations. Charges omitted for clarity.

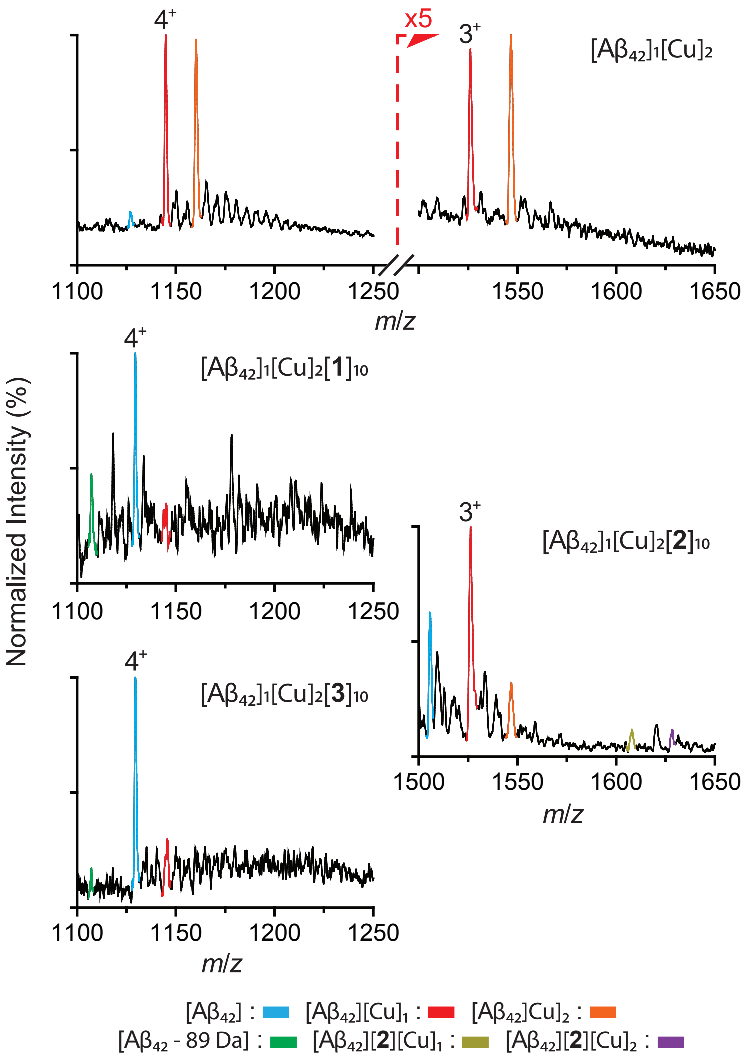


**Supplementary Figure 7 | ESI–MS analyses of A$\beta_{42}$ incubated with Cu(II) and 1-3.** Compared against metal-containing and ligand-free data, **1-3** are shown to be capable of metal-dependent interactions with monomeric A$\beta_{42}$ ([A$\beta_{42}$] = 40 μM; [Cu(II)] = 80 μM; [**1-3**] = 400 μM). Both **1** and **3** promote the formation of Aβ mass loss product 89 Da lighter than the metal-free peptide, consistent with our A$\beta_{40}$ data. **2** is observed to produce stable ternary complexes comprising the molecule, A$\beta_{42}$, and one to two Cu(II). Whilst studies with **4** were attempted, the greatly increased aggregation kinetics and additional metal-associated chemical noise provided by the copper ions prevented successful completion. Differences in the charge state depicted in the figure are used to best represent the complexes observed.

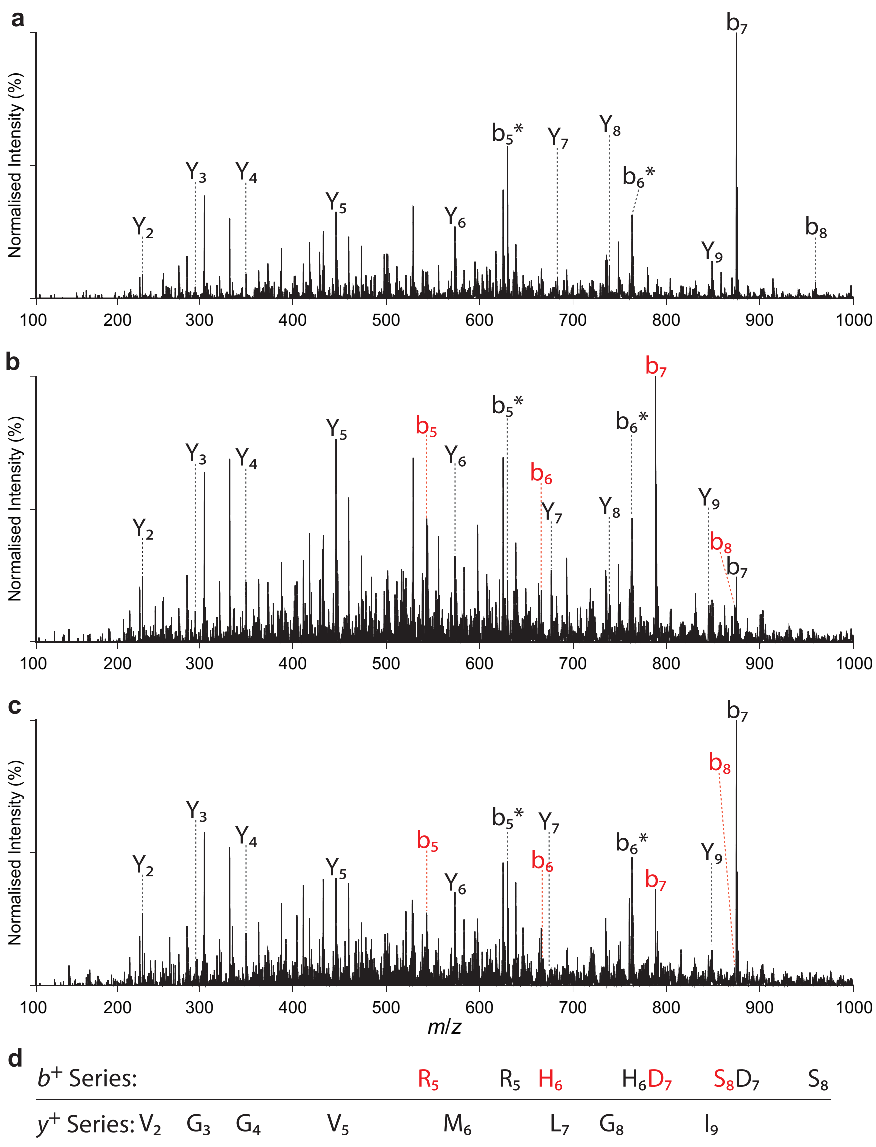


**Supplementary Figure 8 | Tandem mass spectrometry sequencing studies of the metal-dependent chemical modification observed in 1 and 3.** Tandem mass spectrometry ($MS^2$) analysis supports that both (**b**) **1** and (**c**) **3** are capable of producing a metal-dependent chemical modification that leads to a mass loss of 89 Da. In both instances data support that the chemical modification is contained within the first five residues of the Aβ N-terminus (D1, A2, E3, F4, and R5), and is consistent with previously published data[1]. Data are shown against control $A\beta_{40}$ $MS^2$ sequencing data (**a**) acquired under the same conditions. (**d**) Singly charged *b* and *y* ions are shown with the sequence fragments containing the identified mass loss highlighted in red. Ions highlighted with an asterisk indicate overlapping $b^+$/internal sequence fragments irresolvable due to resolution limitations of the instrument. Complementing the above, a list of all peptides identified in our $MS^2$ data is presented in Supplementary Table 5.

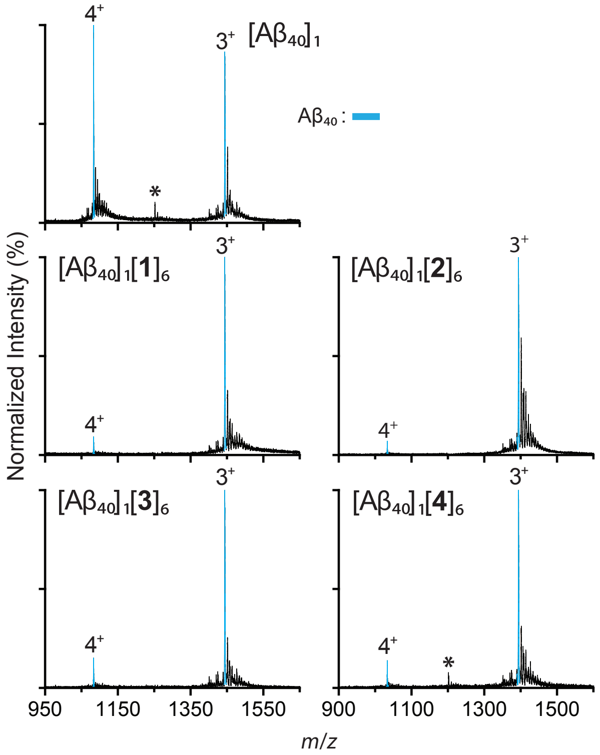


**Supplementary Figure 9 | ESI–MS analyses of Aβ$_{40}$ incubated with 1-4 under copper-free conditions.** Data support that none of the compounds is capable of binding Aβ$_{40}$ in the absence of Cu(II) within a time frame consistent with those presented in Fig. 5 ([Aβ$_{40}$] = 18 μM; [**1**-**4**] = 120 μM). The * represents an adduct refractory to the preparatory methods employed to prepare the peptides for analysis.

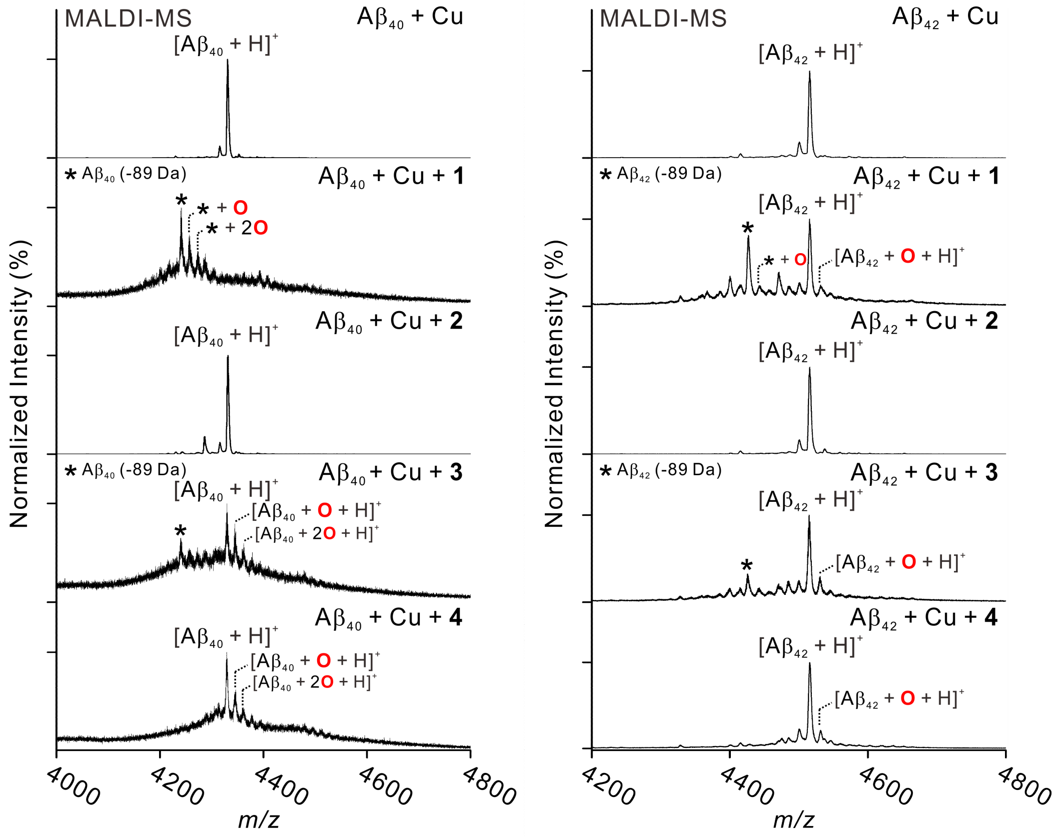


**Supplementary Figure 10 | MALDI–MS spectra of Aβ$_{40}$ (left) or Aβ$_{42}$ (right) incubated with 1-4 in the presence of Cu(II) for 24 h.** With the addition of **1** and **3**, the truncated Aβ$_{40}$ (loss of 89 Da, indicated with an asterisk) appears, which implies that **1** and **3** have similar interactions to **L2-b** with Aβ[1]. Oxidized products were observed in the presence of **1**, **3**, and **4**.

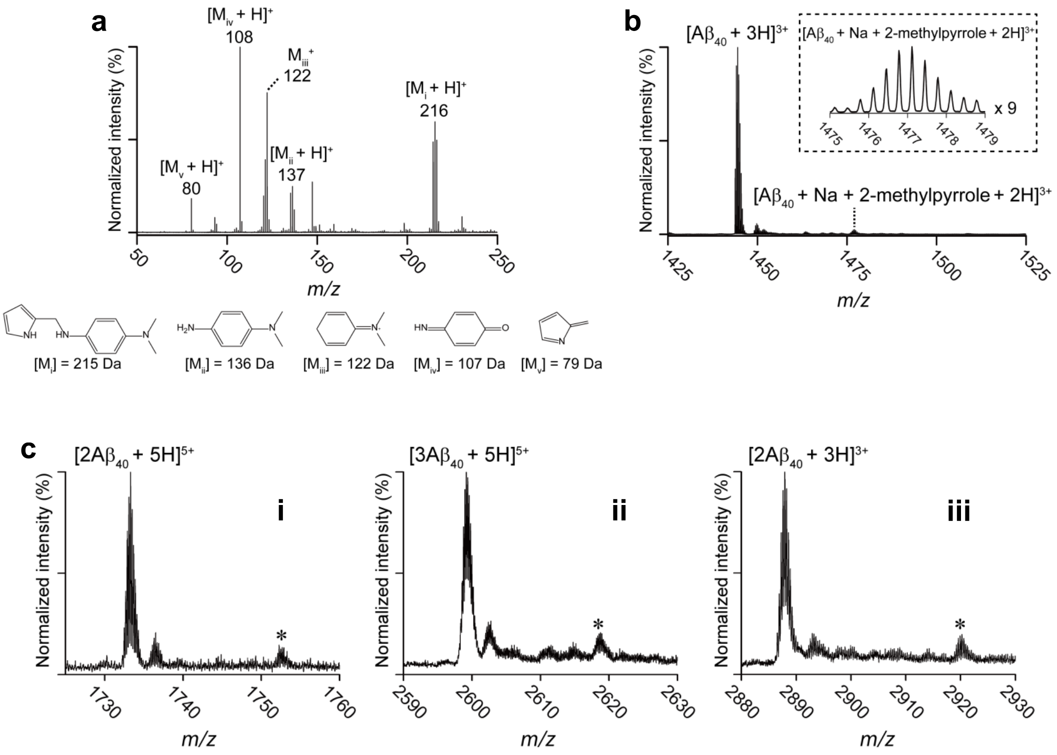


**Supplementary Figure 11 | ESI–MS analyses of 4 with Aβ$_{40}$ under metal-free conditions.** ESI–MS spectra for analyzing the resultant **4** (**a**; from 50 to 250 Da) and monomeric +3-charged Aβ (**b**; from 1425 to 1525 Da) upon incubation of the compound with Aβ for 6 h. (**a**) **4** is observed to be cleaved into 2-methyl-*1H*-pyrrole (81 Da) and *N,N*-dimethyl-*p*-phenylenediamine (M$_{ii}$), which could be further transformed into *p*-benzoquinoneimine (M$_{iv}$). (**b**) The indicated peptide ion has an increase of 103 Da in mass from +3-charged Aβ$_{40}$, which corresponds to an adduct of Aβ with a sodium ion and 2-methylpyrrole (81 Da). (**c**) ESI–MS spectra for oligomeric Aβ species (**i**, +5-charged dimer; **ii**, +5-charged trimer; **iii**, +3-charged dimer) in the presence of **4**. The asterisk denotes the ion composed of Aβ species, 2-methylpyrrole, and sodium ion [for example, in the case of +5-charged dimer, 2Aβ$_{40}$ + 2-methylpyrrole (81 Da) + Na + 4H].

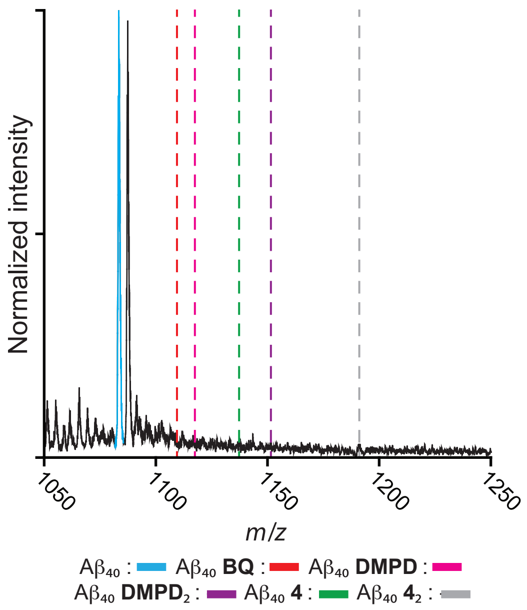


**Supplementary Figure 12 | Mass spectrometric analysis of A$\beta_{40}$ incubated with 4 for 24 h.** Data shown depict incubations of A$\beta_{40}$ with **4** after 24 h at a ratio of 1:25 (peptide concentration, 25 μM). Data support that an interaction between the ligand and monomeric Aβ was not observed over this time span. Data further account for any potential interactions between Aβ and **DMPD** or benzoquinone (**BQ**). The projected *m*/*z* locations of these peaks are shown using dashed lines.

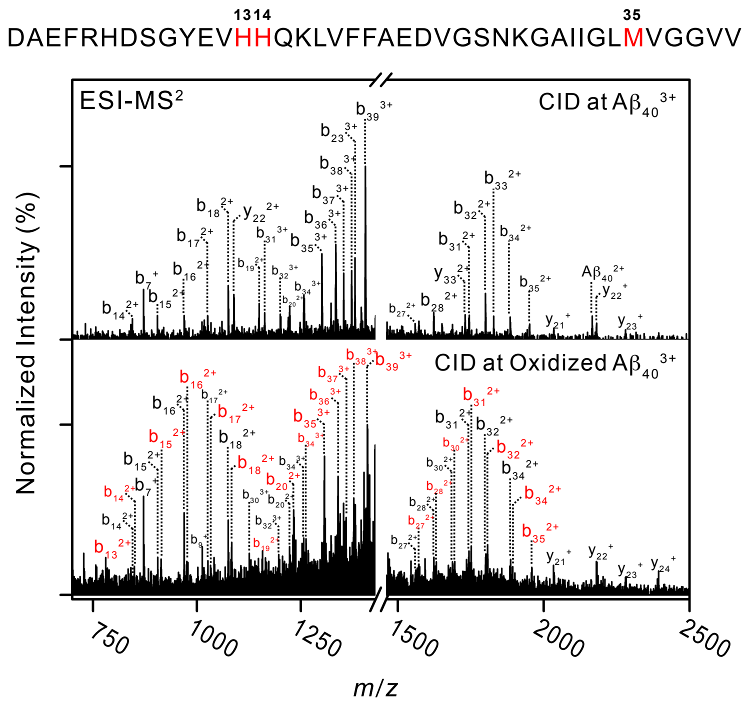


**Supplementary Figure 13 | ESI–MS$^2$ analysis at the +3-charged Aβ (*m*/*z* 1444) and oxidized Aβ (*m*/*z* 1449) found in the presence of Cu(II) and 4.** $b_n^{z+}$ indicates the *z*-charged N-terminal fragment ions including 1 to $n^{th}$ amino acids. y ions denote the C-terminal fragments. The residue (methionine) is known to be readily oxidized and transformed into the methionine sulfoxide or methionine sulfone[2]. In addition, the histidine residue is another plausible oxidation site in Aβ[3], which can form 2-oxo-histidine[4]. Fragments larger than $b_{35}$ only exist as the oxidized form. From $b_{13}$ to $b_{34}$, most ions are found in both unoxidized and oxidized forms. These results indicate that there are several oxidation sites other than M35, which are probably H13 and H14.

**Supplementary Table 1 | Calculated and measured BBB permeability parameters for 1-4.**

| Parameters | 1 | 2 | 3 | 4 | Lipinski's rules and others |
|---|---|---|---|---|---|
| MW[a] | 199 | 244 | 277 | 215 | ≤ 450 |
| *c*LogP[b] | 1.96 | 1.73 | 3.14 | 1.86 | ≤ 5.00 |
| HBA[c] | 3 | 4 | 3 | 3 | ≤ 10 |
| HBD[d] | 3 | 1 | 1 | 2 | ≤ 5 |
| PSA (Å$^2$)[e] | 50.9 | 43.4 | 28.2 | 31.1 | ≤ 90 |
| logBB[f] | −0.563 | −0.249 | 0.191 | −0.047 | < −1.0 poorly distributed in the brain |
| $-\log P_e$[g] | 5.0(1) | 4.2(8) | 4.2(2) | 4.9(0) | $-\log P_e < 5.4$ (CNS+); $-\log P_e > 5.7$ (CNS-) |
| CNS +/− prediction | CNS+ | CNS+ | CNS+ | CNS+ | |

[a] MW, molecular weight; [b] *c*logP, calculated log of water–octanol partition coefficient; [c] HBA, hydrogen bond acceptor; [d] HBD, hydrogen bond donor; [e] PSA, polar surface area; [f] logBB = −0.0148 × PSA + 0.152 × *c*logP × 0.130. [g] Determined using the parallel artificial membrane permeability assay adapted for BBB (PAMPA-BBB).

**Supplementary Table 2 | Changes in the body weight (gram) of the 5×FAD AD model mice during the experimental period[a].**

| Gender of animals | Treatment | Day | | | | | | | | | | |
|---|---|---|---|---|---|---|---|---|---|---|---|---|
| | | 1 | 3 | 6 | 9 | 12 | 15 | 18 | 21 | 24 | 27 | 30 |
| Male | Vehicle (n = 5) | 28.5 ± 0.8* | 28.0 ± 0.8 | 27.0 ± 0.8 | 27.5 ± 0.8 | 27.0 ± 0.6 | 27.8 ± 0.8 | 27.3 ± 0.8 | 26.8 ± 0.7 | 26.8 ± 0.7 | 27.0 ± 0.7 | 26.8 ± 0.3 |
| | **1** (n = 5) | 28.0 ± 0.8 | 27.6 ± 0.8 | 27.6 ± 0.8 | 27.8 ± 0.7 | 27.4 ± 0.7 | 27.8 ± 0.7 | 28.0 ± 0.7 | 27.8 ± 0.8 | 27.6 ± 0.7 | 28.0 ± 0.9 | 27.0 ± 0.7 |
| | *P* value | 0.6796 | 0.7265 | 0.5871 | 0.7902 | 0.6596 | > 0.9999 | 0.5340 | 0.3772 | 0.4231 | 0.4103 | 0.7972 |
| Female | Vehicle (n = 4) | 20.3 ± 1.0 | 20.6 ± 1.0 | 21.6 ± 0.7 | 20.6 ± 0.7 | 20.6 ± 0.7 | 20.9 ± 1.0 | 21.6 ± 0.9 | 21.8 ± 0.6 | 21.4 ± 0.3 | 21.3 ± 0.7 | 21.8 ± 0.3 |
| | **1** (n = 5) | 20.8 ± 0.6 | 20.8 ± 0.7 | 20.8 ± 0.4 | 21.0 ± 0.6 | 21.2 ± 0.5 | 20.8 ± 0.6 | 21.2 ± 0.8 | 21.4 ± 0.7 | 21.4 0.25± 0.7 | 21.8 ± 0.6 | 21.6 ± 0.7 |
| | *P* value | 0.6703 | 0.8703 | 0.3225 | 0.6505 | 0.4958 | 0.9276 | 0.7435 | 0.6860 | > 0.9999 | 0.5983 | 0.8102 |

[a] There is no difference in the body weight between vehicle- and **1** (1 mg/kg/day)-treated 5×FAD mice throughout the experimental 30 day period.

*Mean ± s.e.m.

**Supplementary Table 3 | Rates of transformation and half lives of 1-4 in the presence and absence of Cu(II).**

| | Metal-free | | + Cu(II) | |
|---|---|---|---|---|
| **Compound** | ***k*** **(min$^{-1}$)**[a] | $t_{1/2}$ **(min)**[b] | ***k*** **(min$^{-1}$)**[a] | $t_{1/2}$ **(min)**[b] |
| **1** | –[c] | –[e] | 0.09 ± 0.03 | 8 ± 3 |
| **2** | –[c] | –[e] | –[c] | –[e] |
| **3** | –[c] | –[e] | 0.013 ± 0.001 | 53 ± 3 |
| **4** | 0.016 ±0.004 | 43 ± 5 | –[d] | < 1 |

[a] Rate of decay of the absorbance peak at 250, 384, and 400 nm for **4**, [Cu(II) + **1**], and [Cu(II) + **2**], respectfully. [b] Half life of the absorbance peak in minutes. [c] Spectral changes were too slow to accurately measure the rate during the duration of the experiment. [d] Decay of compound occurred too rapidly to measure in the experiment conditions ([**1-4**] = 50 μM; [Cu] = 25 μM; 25 μM HEPES, pH 7.4, 150 μM NaCl; 37 °C). [e] No noticeable spectral changes were observed over 5 h.

**Supplementary Table 4 | Calculated collision cross section (CCS) from IM–MS analysis.**

| | Conformational Species (Å$^2$) | | | | |
|---|---|---|---|---|---|
| | 1 | 2 | 3 | 4 | 5 |
| Aβ$_{40}$ | 658.6 +/- 30.6 | 721.8 +/- 31.4 | X | X | X |
| Aβ$_{40}$ Cu | 658.7 +/- 28.8 | 727.3 +/- 31.6 | 782.3 +/- 34.7 | X | X |
| Aβ$_{40}$ Cu$_2$ | 656.9 +/- 32.5 | 732.8 +/- 31.9 | 786.4 +/- 35.6 | X | X |
| Aβ$_{40}$ **2** Cu | X | 717.1 +/- 31.3 | X | 682.9 +/- 32.5 | 760.3 +/- 33.0 |
| Aβ$_{40}$ **2** Cu$_2$ | X | X | X | 683.7 +/- 36.3 | 761.2 +/- 40.8 |

Calculated CCS data are given for the presented stoichiometries and have been calculated using established methods[5,6]. These data suggest the existence of five different conformational species across all ligand stoichiometries observed. Two of these are observed only with **2**. Data are the average of six repeats with errors reported as a function of least square analysis.

**Supplementary Table 5. Products of collision induced fragmentation identified.**

| Products of collision induced dissociation | Ion species observed | | | | |
|---|---|---|---|---|---|
| b sequence ions | z | *m/z* | Apo | Derivative 1 modification | Derivative 3 modification |
| $b_4$ | 1 | 463.1823 | No | No | No |
| $b_5^{‡}$ | 1 | 619.2835 | Yes | No | Yes |
| $b_6^{*}$ | 1 | 756.3424 | Yes | Yes | Yes |
| $b_7$ | 1 | 871.3693 | Yes | Yes | Yes |
| $b_8$ | 1 | 958.4013 | Yes | No | No |
| $b_9$ | 1 | 1015.4228 | Yes | No | No |
| $b_{10}$ | 1 | 1178.4861 | Yes | No | No |
| $b_{11}$ | 1 | 1307.5287 | Yes | No | Yes |
| $b_{12}$ | 1 | 1406.5971 | No | No | No |
| $b_8$ | 2 | 479.7043 | No | No | No |
| $b_9$ | 2 | 508.215 | Yes | No | No |
| $b_{10}$ | 2 | 589.7467 | No | No | No |
| $b_{11}$ | 2 | 654.268 | Yes | No | Yes |
| $b_{12}$ | 2 | 703.8022 | Yes | Yes | Yes |
| $b_{13}$ | 2 | 772.3317 | Yes | No | Yes |
| $b_{14}$ | 2 | 840.8611 | Yes | No | Yes |
| $b_{15}$ | 2 | 904.8904 | Yes | No | Yes |
| $b_{16}$ | 2 | 968.9379 | Yes | No | Yes |
| $b_{17}$ | 2 | 1025.4799 | Yes | No | Yes |
| $b_{18}$ | 2 | 1075.0141 | Yes | No | Yes |
| $b_{19}$ | 2 | 1148.5483 | Yes | No | Yes |
| $b_{20}$ | 2 | 1222.0825 | Yes | No | Yes |
| $b_{21}$ | 2 | 1257.6011 | Yes | No | Yes |
| $b_{22}$ | 2 | 1322.1224 | Yes | No | Yes |
| $b_{23}$ | 2 | 1379.6359 | Yes | Yes | Yes |
| $b_{24}$ | 2 | 1429.1701 | Yes | No | Yes |
| $b_{25}$ | 2 | 1457.6808 | Yes | Yes | Yes |
| $b_{26}$ | 2 | 1501.1968 | Yes | No | No |
| $b_{27}$ | 2 | 1558.2183 | Yes | No | No |
| $b_{28}$ | 2 | 1622.2658 | Yes | No | No |
| $b_{29}$ | 2 | 1650.7765 | No | No | No |
| $b_{30}$ | 2 | 1686.2951 | No | No | No |
| $b_{31}$ | 2 | 1742.8371 | Yes | No | No |
| $b_{32}$ | 2 | 1799.3791 | Yes | No | No |
| $b_{33}$ | 2 | 1827.8898 | No | No | No |

| | | | | | |
|---|---|---|---|---|---|
| $b_{34}$ | 2 | 1884.4319 | Yes | No | No |
| $b_{35}$ | 2 | 1949.9521 | No | No | No |
| $b_{26}$ | 3 | 1001.1336 | Yes | No | No |
| $b_{27}$ | 3 | 1039.1479 | Yes | No | No |
| $b_{28}$ | 3 | 1081.8463 | Yes | No | No |
| $b_{29}$ | 3 | 1100.8534 | Yes | No | No |
| $b_{30}$ | 3 | 1124.5325 | Yes | No | No |
| $b_{31}$ | 3 | 1162.2271 | Yes | No | No |
| $b_{32}$ | 3 | 1199.9218 | Yes | No | No |
| $b_{33}$ | 3 | 1218.929 | Yes | No | No |
| $b_{34}$ | 3 | 1256.6237 | Yes | No | No |
| $b_{35}$ | 3 | 1300.3038 | Yes | No | No |
| $b_{36}$ | 3 | 1333.3266 | Yes | No | No |
| $b_{37}$ | 3 | 1352.3338 | Yes | No | No |
| $b_{38}$ | 3 | 1371.341 | Yes | No | No |
| $b_{39}$ | 3 | 1404.3638 | Yes | No | No |
| Modified $b_4$ | 1 | 374.1261 | No | No | No |
| Modified $b_5$ | 1 | 530.2272 | No | Yes | Yes |
| Modified $b_6$ | 1 | 667.2861 | No | Yes | Yes |
| Modified $b_7$ | 1 | 782.3131 | No | Yes | Yes |
| Modified $b_8$ | 1 | 869.3451 | No | Yes | Yes |
| Modified $b_9$ | 1 | 926.3666 | No | No | No |
| Modified $b_{11}$ | 2 | 609.7399 | No | Yes | Yes |
| Modified $b_{12}$ | 2 | 659.2741 | No | Yes | No |
| Modified $b_{13}$ | 2 | 727.8035 | No | Yes | Yes |
| Modified $b_{14}$ | 2 | 796.333 | No | Yes | Yes |
| Modified $b_{15}$ | 2 | 860.3623 | No | Yes | Yes |
| Modified $b_{16}$ | 2 | 924.4098 | No | Yes | Yes |
| Modified $b_{17}$ | 2 | 980.9518 | No | Yes | Yes |
| Modified $b_{18}$ | 2 | 1030.486 | No | Yes | Yes |
| Modified $b_{19}$ | 2 | 1104.0202 | No | Yes | Yes |
| Modified $b_{20}$ | 2 | 1177.5544 | No | Yes | Yes |
| Modified $b_{21}$ | 2 | 1213.073 | No | Yes | Yes |
| Modified $b_{22}$ | 2 | 1277.5943 | No | Yes | No |
| Modified $b_{23}$ | 2 | 1335.1077 | No | Yes | Yes |
| Modified $b_{24}$ | 2 | 1384.642 | No | Yes | No |
| Modified $b_{25}$ | 2 | 1413.1527 | No | Yes | Yes |
| Modified $b_{26}$ | 2 | 1456.6687 | No | No | No |
| Modified $b_{27}$ | 2 | 1513.6902 | No | Yes | No |
| Modified $b_{28}$ | 2 | 1577.7376 | No | Yes | No |
| Modified $b_{29}$ | 2 | 1606.2484 | No | No | No |

| | | | | | |
|---|---|---|---|---|---|
| Modified $b_{30}$ | 2 | 1641.7669 | No | Yes | Yes |
| Modified $b_{31}$ | 2 | 1698.309 | No | Yes | Yes |
| Modified $b_{32}$ | 2 | 1754.851 | No | No | No |
| Modified $b_{33}$ | 2 | 1783.3617 | No | Yes | No |
| Modified $b_{34}$ | 2 | 1839.9038 | No | Yes | Yes |
| Modified $b_{27}$ | 3 | 1009.4625 | No | No | No |
| Modified $b_{28}$ | 3 | 1052.1609 | No | Yes | No |
| Modified $b_{29}$ | 3 | 1071.168 | No | Yes | Yes |
| Modified $b_{30}$ | 3 | 1094.847 | No | Yes | Yes |
| Modified $b_{31}$ | 3 | 1132.5417 | No | Yes | Yes |
| Modified $b_{32}$ | 3 | 1170.2364 | No | Yes | Yes |
| Modified $b_{33}$ | 3 | 1189.2436 | No | Yes | Yes |
| Modified $b_{34}$ | 3 | 1226.9383 | No | Yes | Yes |
| Modified $b_{35}$ | 3 | 1270.6184 | No | Yes | Yes |
| Modified $b_{36}$ | 3 | 1303.6412 | No | Yes | Yes |
| Modified $b_{37}$ | 3 | 1322.6484 | No | No | No |
| Modified $b_{38}$ | 3 | 1341.6555 | No | Yes | Yes |
| Modified $b_{39}$ | 3 | 1374.6783 | No | Yes | Yes |

| Products of collision induced dissociation | Ion species observed | | | | |
|---|---|---|---|---|---|
| y sequence ions | z | *m*/*z* | Apo | Derivative 1 modification | Derivative 3 modification |
| $y_2$ | 1 | 217.1547 | Yes | Yes | Yes |
| $y_3$ | 1 | 274.1761 | Yes | Yes | Yes |
| $y_4$ | 1 | 331.1976 | Yes | Yes | Yes |
| $y_5$ | 1 | 430.266 | Yes | Yes | Yes |
| $y_6$ | 1 | 561.3065 | Yes | Yes | Yes |
| $y_7$ | 1 | 674.3906 | Yes | Yes | Yes |
| $y_8$ | 1 | 731.412 | Yes | Yes | Yes |
| $y_9$ | 1 | 844.4961 | Yes | Yes | Yes |
| $y_{10}$ | 1 | 957.5802 | No | No | No |
| $y_{20}$ | 2 | 943.5062 | No | No | No |
| $y_{21}$ | 2 | 1017.0404 | Yes | Yes | Yes |
| $y_{22}$ | 2 | 1090.5746 | No | Yes | Yes |
| $y_{23}$ | 2 | 1140.1088 | No | No | No |
| $y_{31}$ | 2 | 1657.3737 | No | No | No |
| $y_{32}$ | 2 | 1685.8845 | Yes | Yes | Yes |
| $y_{33}$ | 2 | 1729.4005 | Yes | Yes | Yes |
| $y_{34}$ | 2 | 1786.9139 | Yes | Yes | Yes |

| | | | | | |
|---|---|---|---|---|---|
| $y_{35}$ | 2 | 1855.4434 | Yes | No | No |
| $y_{36}$ | 2 | 1933.494 | No | No | No |
| $y_{31}$ | 3 | 1105.2516 | No | No | No |
| $y_{32}$ | 3 | 1124.2587 | Yes | Yes | Yes |
| $y_{33}$ | 3 | 1153.2694 | Yes | No | No |
| $y_{34}$ | 3 | 1191.6117 | Yes | No | No |
| $y_{35}$ | 3 | 1237.298 | No | No | No |

| Products of collision induced dissociation | Ion species observed | | | | |
|---|---|---|---|---|---|
| Internal sequence ions | z | *m/z* | Apo | Derivative 1 modification | Derivative 3 modification |
| KGA-NH3 and QK-NH3 | 1 | 240.1343 | Yes | Yes | Yes |
| KGA and QK | 1 | 257.1608 | Yes | Yes | Yes |
| HQ | 1 | 266.1248 | Yes | Yes | Yes |
| VGGV-28 | 1 | 285.1921 | Yes | Yes | Yes |
| GLM | 1 | 302.1533 | Yes | Yes | Yes |
| VGGV | 1 | 313.187 | Yes | Yes | Yes |
| MVGG | 1 | 345.1591 | Yes | Yes | Yes |
| AIIG and GAII | 1 | 355.234 | Yes | Yes | Yes |
| KGAI and QKL | 1 | 370.2449 | Yes | Yes | Yes |
| GSNK and SNKG | 1 | 387.1987 | No | Yes | Yes |
| VFF | 1 | 394.2125 | Yes | Yes | Yes |
| HQF | 1 | 394.2197 | Yes | Yes | Yes |
| GLMV and LMVG | 1 | 401.2217 | Yes | Yes | Yes |
| GAIIG | 1 | 412.2554 | Yes | Yes | Yes |
| GSNKG-28 | 1 | 416.2252 | Yes | Yes | Yes |
| MVGGV-28 | 1 | 416.2326 | Yes | Yes | Yes |
| GSNKG | 1 | 444.2201 | Yes | Yes | Yes |
| MVGGV | 1 | 444.2275 | Yes | Yes | Yes |
| QKLV-$NH_3$ | 1 | 452.2867 | Yes | Yes | Yes |
| VGSNK-28 | 1 | 458.2722 | Yes | Yes | Yes |
| KGAII | 1 | 483.3289 | Yes | Yes | Yes |
| NKGAI | 1 | 484.2878 | No | No | Yes |
| VGSNK | 1 | 486.2671 | Yes | Yes | Yes |
| GAIIGL-28 | 1 | 497.3446 | Yes | Yes | Yes |
| HQKL | 1 | 507.3038 | Yes | Yes | Yes |
| IGLMV | 1 | 514.3058 | No | No | Yes |
| GSNKGA | 1 | 515.2572 | Yes | Yes | Yes |
| GLMVGG | 1 | 515.2646 | Yes | Yes | Yes |

| | | | | | |
|---|---|---|---|---|---|
| GAIIGL | 1 | 525.3395 | Yes | Yes | Yes |
| SNKGAI-28 | 1 | 543.3249 | Yes | Yes | Yes |
| IGLMVG-28 | 1 | 543.3323 | Yes | Yes | Yes |
| SNKGAI | 1 | 571.3198 | Yes | Yes | Yes |
| IGLMVG | 1 | 571.3272 | Yes | Yes | Yes |
| VGSNKGA-28 | 1 | 586.3307 | Yes | Yes | Yes |
| GLMVGGV-28 | 1 | 586.3381 | Yes | Yes | Yes |
| FAEDVG-28 | 1 | 591.2773 | Yes | Yes | Yes |
| HQKLV | 1 | 606.3722 | Yes | Yes | Yes |
| VGSNKGA | 1 | 614.3257 | Yes | Yes | Yes |
| GLMVGGV | 1 | 614.333 | Yes | Yes | Yes |
| FAEDVG‡ | 1 | 619.2722 | Yes | Yes | Yes |
| GSNKGAI | 1 | 628.3413 | Yes | Yes | Yes |
| IGLMVGG | 1 | 628.3487 | Yes | Yes | Yes |
| GAIIGLM | 1 | 656.38 | Yes | Yes | Yes |
| IIGLMVG | 1 | 684.4113 | Yes | Yes | Yes |
| VGSNKGAI-28 | 1 | 699.4148 | Yes | Yes | Yes |
| IGLMVGGV-28 | 1 | 699.4222 | Yes | Yes | Yes |
| VGSNKGAI | 1 | 727.4097 | Yes | Yes | Yes |
| IGLMVGGV | 1 | 727.4171 | Yes | Yes | Yes |
| GSNKGAII and SNKGAIIG | 1 | 741.4254 | Yes | Yes | Yes |
| IIGLMVGG | 1 | 741.4328 | Yes | Yes | Yes |
| HQKLVF | 1 | 753.4406 | Yes | Yes | Yes |
| AEFRHD* | 1 | 756.3424 | Yes | Yes | Yes |
| GSNKGAIIG | 1 | 798.4468 | Yes | Yes | Yes |
| VGSNKGAII | 1 | 840.4938 | Yes | Yes | Yes |
| IIGLMVGGV | 1 | 840.5012 | Yes | Yes | Yes |
| SNKGAIIGL | 1 | 854.5094 | Yes | No | No |
| KGAIIGLMV-$NH_3$ | 1 | 866.5168 | Yes | Yes | No |
| KGAIIGLMV and AIIGLMVGGV-28 | 1 | 883.5434 | Yes | Yes | Yes |
| GSNKGAIIGL | 1 | 911.5309 | Yes | No | No |
| AIIGLMVGGV | 1 | 911.5383 | Yes | No | No |
| NKGAIIGLMV and KGAIIGLMVGG | 1 | 997.5863 | Yes | No | No |

All $b^+$ ions with the prefix 'Modified' represent those containing the observed mass loss of 89.2 Da. * Due to instrumental resolution limits peaks highlighted may represent either $b_6^+$ or an internal $A_2EFRHD^+$ fragment. ‡ Due to instrumental resolution limits peaks highlighted may represent either $b_5^+$ or an internal $F_{20}AEDVG$ fragment.

## Supplementary Methods

**Materials and methods.** All reagents were purchased from commercial suppliers and used as received unless otherwise noted. A$\beta_{40}$ and A$\beta_{42}$ (the sequence of A$\beta_{42}$: DAEFRHDSGYEVHHQKLVFFAEDVGSNKGAIIGLMVGGVVIA) were purchased from Anaspec Inc. (Fremont, CA, USA). Trace metals were removed from buffers and solutions used in A$\beta$ experiments by treating with Chelex overnight (Sigma-Aldrich, St. Louis, MO, USA). Optical spectra were recorded on an Agilent 8453 UV-visible spectrophotometer. Absorbance values for biological assays, including cell viability and antioxidant assays, were measured on a Molecular Devices SpectraMax 190 microplate reader (Sunnyvale, CA, USA). $^{1}$H and $^{13}$C NMR spectra were recorded using a 400 MHz Agilent NMR spectrometer.

**Preparation of 4-nitro-*N*-(pyridin-2-ylmethyl)aniline.** 4-Nitro-*N*-(pyridin-2-ylmethyl)aniline was synthesized using previously reported methods with modifications[7]. To a flame-dried flask equipped with a stir bar and a reflux condenser under $N_2$ (g), 1-fluoro-4-nitrobenzene (231 μL, 2.2 mmol) and *N*,*N*-diisopropylethylamine (836 μL, 4.8 mmol) were added to DMF (25 mL) followed by the introduction of 2-(aminomethyl)pyridine (247 μL, 2.4 mmol) at room temperature. The resulting solution was heated to 70 °C. After 24 h, the brown solution was added to water (75 mL) and extracted with EtOAc (3 x 75 mL). The organic layers were washed with water (2 x 75 mL) and brine (75 mL), dried with $MgSO_4$, and concentrated under vacuum. The resulting residue was then purified on a silica column (25% to 100% EtOAc in hexanes) yielding a yellow solid (0.29 g, 58%) [TLC (EtOAc:hexanes = 50:50 (v/v)): $R_f$ = 0.25]. $^{1}$H

NMR [400 MHz, $CD_2Cl_2$, δ (ppm)]: 8.56 (d, 1H, *J* = 8 Hz), 8.09 (d, 2H, *J* = 8 Hz), 7.71 (t, 1H, *J* = 8 Hz), 7.30 (d, 1H, *J* = 8 Hz), 7.25 (d, 1H, *J* = 4 Hz), 6.68 (d, 2 H, *J* = 8 Hz), 6.04 (s, 1H), 4.53 (d, 2H, *J* = 8 Hz). $^{13}$C NMR [100 MHz, $CD_2Cl_2$, δ (ppm)]: 156.2, 153.5, 149.5, 138.5, 137.2, 126.6, 123.0, 122.1, 111.8, 48.2.

**Preparation of 1.** The compound **1** was purchased from Ryan Scientific (Mt. Pleasant, SC, USA) and recrystallized from $CH_2Cl_2$/hexanes four times. $^1$H NMR [400 MHz, $(CD_3)_2SO$, δ (ppm)]: 8.49 (d, 1H, *J* = 4 Hz), 7.70 (t, 1H, *J* = 4 Hz), 7.35 (d, 1H, *J* = 4 Hz), 7.22 (t, 1H, *J* = 4 Hz), 6.36 (m, 4H), 5.46 (s, 1H), 4.23 (m, 4H). $^{13}$C NMR [100 MHz, $CD_2Cl_2$, δ (ppm)]: 159.58, 149.50, 141.44, 138.65, 136.75, 122.30, 121.99, 116.85, 114.88, 50.69. ESI(+)MS (*m*/z): $[M+H]^+$ Calcd. for $C_{12}H_{14}N_3$, 200.12; found, 200.03.

Additionally, **1** was also synthesized by adapting previously reported methods[8] to reduce 4-nitro-*N*-(pyridin-2-ylmethyl)aniline. To a solution of 4-nitro-*N*-(pyridin-2-ylmethyl)aniline (0.52 g, 2.3 mmol) and *tris*(acetylacetonato)iron(III) (0.024 g, 3 mol%) in ethanol (20 mL) in a round-bottom flask equipped with a stir bar and a reflux condenser, hydrazine hydrate (581 μL, 11 mmol) was added. The solution was then heated under reflux for 2 h and additional 4 equivalents of hydrazine hydrate were introduced. The mixture was allowed to react for an additional 3 h before removing the solvent under vacuum. The resulting brown oil was purified by silica column chromatography (EtOAc (100%) to EtOAc:$Et_3N$ (99%:1%); TLC (EtOAc:$Et_3N$ = 99:1 (v/v)), $R_f$ = 0.20). The HCl salt of the product was then prepared by dissolving in MeOH and adding excess 5 M HCl. The solvent was removed under vacuum and the resulting residue was washed with $Et_2O$ (3 x 5 mL). The residues were dissolved in water (20 mL). The aqueous layer

was washed with $Et_2O$ (3 x 20 mL) and collected. After removing the water under vacuum and recrystallization using MeOH and $Et_2O$, the product was obtained (pale yellow powder, 0.45 g. 85%). $^1H$ NMR [400 MHz, $(CD_3)_2SO$, δ (ppm)]: 9.59 (s, 1H), 8.59 (d, 1H, J = 4 Hz), 7.90 (t, 1H, J = 4 Hz), 7.59 (m, 2H), 7.05 (d, 2H, J = 8 Hz), 6.67 (d, 2H, J = 8 Hz), 4.43 (s, 2H). $^{13}C$ NMR [100 MHz, $(CD_3)_2SO$, δ (ppm)]: 156.0, 147.1, 144.6, 142.9, 125.0, 124.6, 124.0, 120.8, 113.0, 44.8. HRMS (m/z): $[M+H]^+$ Calcd. for $C_{12}H_{14}N_3$, 200.1188; found, 200.1190.

**Preparation of 2.** The compound **2** was purchased from Ryan Scientific and recrystallized from $CH_3CN$ and water three times (off-white powder). $^1H$ NMR [400 MHz, $(CD_3)_2SO$, δ (ppm)]: 8.52 (d, 1H, J = 4 Hz), 7.73 (t, 1H, J = 8 Hz), 7.35 (d, 1H, J = 8 Hz), 7.24 (t, 1H, J = 8 Hz), 6.35 (t, 1H, J = 8 Hz), 5.75 (s, 2H), 5.71 (s, 1H), 4.31 (d, 2H, 4 Hz), 3.61 (s, 6H). $^{13}C$ NMR [100 MHz, $(CD_3)_2SO$, δ (ppm)]: 161.1, 159.8, 150.2, 148.8, 136.6, 122.0, 121.1, 91.1, 88.6, 54.7, 48.5. HRMS (m/z): $[M+H]^+$ Calcd. for $C_{14}H_{17}N_2O_2$, 245.1290; found, 245.1288.

**Preparation of 3.** The compound **3** was purchased from Ryan Scientific and was recrystallized from hot hexanes and washed 5x with cold hexanes (yellow powder). $^1H$ NMR [400 MHz, $(CD_3)_2SO$, δ (ppm)]: 8.29 (d, 1H, J = 8 Hz), 8.00 (d, 1H, J = 8 Hz), 7.93 (d, 1H, J = 8 Hz), 7.74 (t, 1H, J = 8 Hz), 7.56 (m, 2H), 6.57 (m, 4H), 5.91 (t, 1H, J = 4 Hz), 4.47 (d, 2H, J = 8 Hz), 2.67 (s, 6H). $^{13}C$ NMR [100 MHz, $CD_2Cl_2$, δ (ppm)]: 159.9, 148.2, 144.8, 141.0, 136.9, 130.0, 129.4, 128.2, 127.9, 126.6, 120.5, 116.1, 114.7, 51.2, 42.39. HRMS (m/z): $[M+H]^+$ Calcd for $C_{18}H_{20}N_3$, 278.1657, found, 278.1656.

**Preparation of 4.** The compound **4** was purchased from Ukrorgsyntez (Ukraine) and washed with hexanes with one drop of $CH_2Cl_2$ once and hexanes three or four times (dark brown powder). $^1H$ NMR [400 MHz, $(CD_3)_2SO$, δ (ppm)]: 10.65 (s, 1H), 6.60 (m, 4H), 5.91 (s, 2H), 5.09 (s, 1H), 4.07 (d, 2H, J = 8 Hz), 2.70 (s, 6H). $^{13}C$ NMR [100 MHz, $CD_2Cl_2$, δ (ppm)]: 145.3, 140.9, 130.8, 117.6, 115.9, 115.2, 108.7, 106.3, 43.20, 42.27. HRMS (m/z): $[M+H]^+$ Calcd. for $C_{13}H_{18}N_3$, 216.1501; found, 216.1502.

**Supplementary References**


1. Beck, M. W. *et al.* A rationally designed small molecule for identifying an *in vivo* link between metal–amyloid-β complexes and the pathogenesis of Alzheimer's disease. *Chem. Sci.* **6**, 1879–1886 (2015).
2. Stadtman, E. R. & Levine, R. L. Protein oxidation. *Ann. N. Y. Acad. Sci.* **899**, 191-208 (2000).
3. Inoue, K., Garner, C., Ackermann, B. L., Oe, T. & Blair, I. A. Liquid chromatography/tandem mass spectrometry characterization of oxidized amyloid beta peptides as potential biomarkers of Alzheimer's disease. *Rapid Commun. Mass Spectrom.* **20**, 911-918 (2006).
4. Uchida, K. Histidine and lysine as targets of oxidative modification. *Amino Acids* **25**, 249-257 (2003).
5. Ruotolo, B. T.; Benesch, J. L. P.; Sandercock, A. M.; Hyung, S.-J.; Robinson, C. V. Ion mobility–mass spectrometry analysis of large protein complexes. *Nat. Protoc.* **3**, 1139-1152 (2008).

6. Bush, M. F.; Hall, Z.; Giles, K.; Hoyes, J.; Robinson, C. V.; Ruotolo, B. T. Collision cross sections of proteins and their complexes: a calibration framework and database for gas-phase structural biology. *Anal. Chem.* **82**, 9557−9565 (2010).

7. Jones, C. K. *et al*. Discovery, synthesis, and structure–activity relationship development of a series of *N*-4-(2,5-dioxopyrrolidin-1-yl)phenylpicolinamides (VU0400195, ML182): characterization of a novel positive allosteric modulator of the metabotropic glutamate receptor 4 (mGlu4) with oral efficacy in an antiparkinsonian animal model. *J. Med. Chem.* **54**, 7639−7647 (2011).

8. Sharma, U. *et al.* Phosphane-free green protocol for selective nitro reduction with an iron-based catalyst. *Chem. Eur. J.* **17**, 5903−5907 (2011).
